# Cannabinoids Modulate Neuronal Activity and Cancer by CB1 and CB2 Receptor-Independent Mechanisms

**DOI:** 10.3389/fphar.2017.00720

**Published:** 2017-10-10

**Authors:** Ken Soderstrom, Eman Soliman, Rukiyah Van Dross

**Affiliations:** ^1^Department of Pharmacology and Toxicology, Brody School of Medicine, East Carolina University, Greenville, NC, United States; ^2^Department of Pharmacology and Toxicology, Zagazig University, Zagazig, Egypt; ^3^Center for Health Disparities, East Carolina University, Greenville, NC, United States

**Keywords:** cannabinoid, cannabinoid receptor-independent, central nervous system, Cancer, experimental therapeutics

## Abstract

Cannabinoids include the active constituents of *Cannabis* or are molecules that mimic the structure and/or function of these *Cannabis*-derived molecules. Cannabinoids produce many of their cellular and organ system effects by interacting with the well-characterized CB1 and CB2 receptors. However, it has become clear that not all effects of cannabinoid drugs are attributable to their interaction with CB1 and CB2 receptors. Evidence now demonstrates that cannabinoid agents produce effects by modulating activity of the entire array of cellular macromolecules targeted by other drug classes, including: other receptor types; ion channels; transporters; enzymes, and protein- and non-protein cellular structures. This review summarizes evidence for these interactions in the CNS and in cancer, and is organized according to the cellular targets involved. The CNS represents a well-studied area and cancer is emerging in terms of understanding mechanisms by which cannabinoids modulate their activity. Considering the CNS and cancer together allow identification of non-cannabinoid receptor targets that are shared and divergent in both systems. This comparative approach allows the identified targets to be compared and contrasted, suggesting potential new areas of investigation. It also provides insight into the diverse sources of efficacy employed by this interesting class of drugs. Obtaining a comprehensive understanding of the diverse mechanisms of cannabinoid action may lead to the design and development of therapeutic agents with greater efficacy and specificity for their cellular targets.

## Introduction

Cannabinoids are a broad and diverse class of drugs that are structurally- or functionally-related to those isolated from *Cannabis* (i.e., are “*Cannabis*-like”). Several structural classes of cannabinoid drugs have been identified, including: phytocannabinoids (related to those derived from plant material); endogenously-produced cannabinoids; and related eicosanoids that regulate vertebrate endocannabinoid signaling systems; synthetic and other types of cannabinoids (many developed seeking modulators of other signaling systems, but found to interact with the classic CB1 and/or CB2 receptors). Classical cannabinoid agonists (structures shown in Table 1) bind to and activate cannabinoid receptors 1 and 2 (CB1, CB2) that modulate signal transduction cascades to produce various physiological and pathological outcomes. The actions of cannabinoids are also regulated by the endocannabinoid system (ECS) which includes enzymes involved in synthesis, uptake and degradation of endogenous cannabinoid ligands, and the CB1 and CB2 receptors. Cannabinoid pharmacology is an active field, and new examples of cannabinoid drugs are identified regularly (Shevyrin et al., 2016) with the primary goal of discovering novel therapeutics.

**Table 1:** Structures of cannabinoids reviewed, summarized by class.

**Endocannabinoids (ECs)**			
**Arachidonic acid-derived ECs**			
AEA 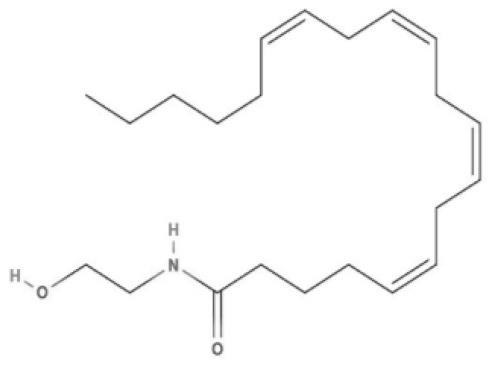	Noladin Ether (2-AG ether) 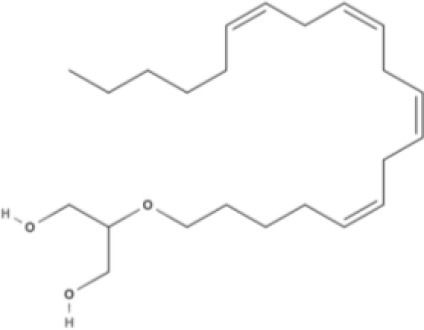	2-AG 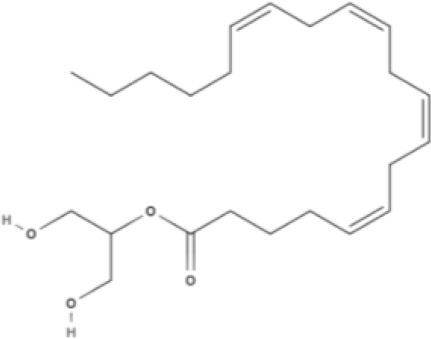	Virodhamine 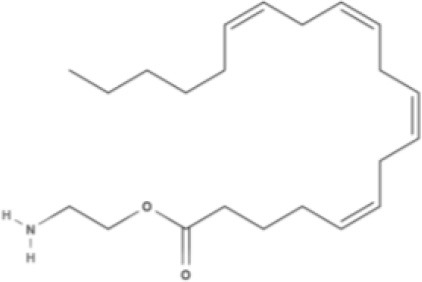
NAGly 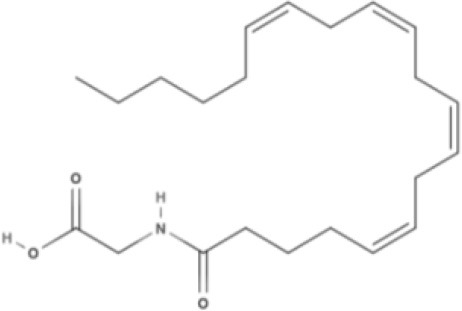	2A-LPA 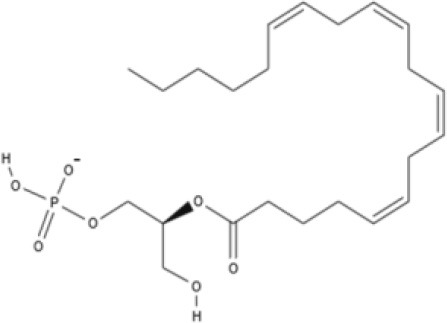	**Oleic acid-derived EC** Oleoylethanolamide (OEA) 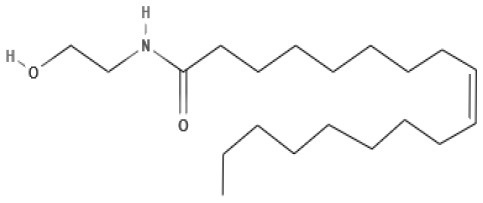	**Palmitic acid-derived EC** Palmitoylethanolamide (PEA) 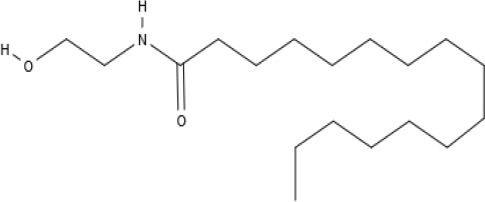
**Other EC** Lysophosphatidylinositol (LPI) 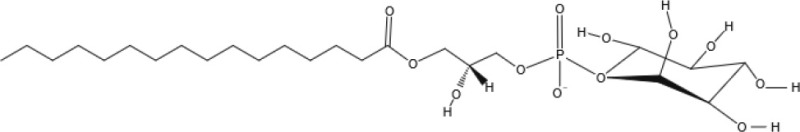			
**Phytocannabinoids**			
**Tricyclic phytocannabinoids**			
THC 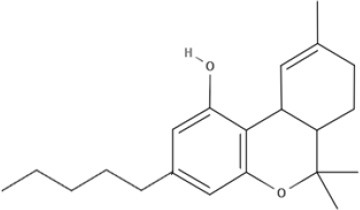	THCA 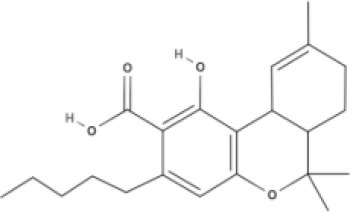	THCV 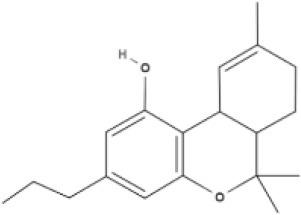	THCVA 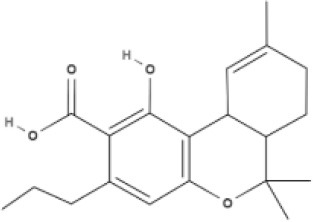
CBN 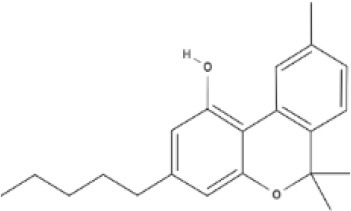			
**Bicyclic phytocannabinoids**			
CBD 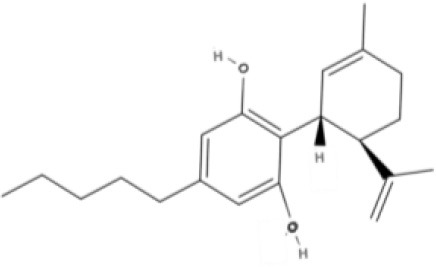	CBDA 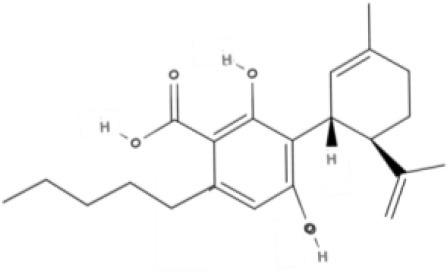	CBDV 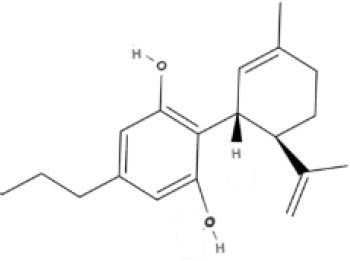	CBDVA 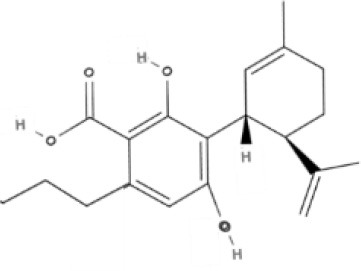
AbCBD 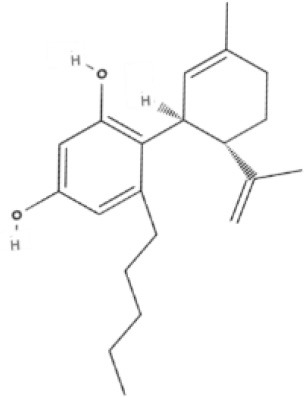			
**Other phytocannabinoids**			
CBG 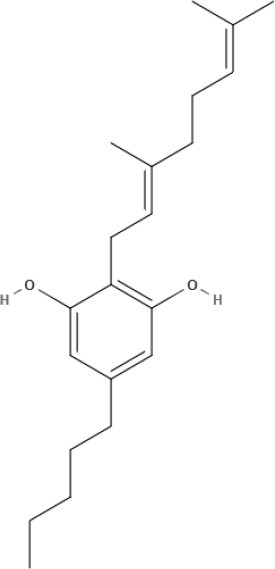	CBGV 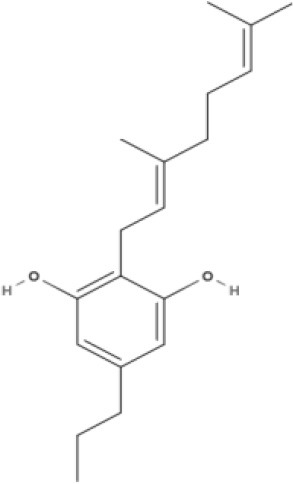	CBGA 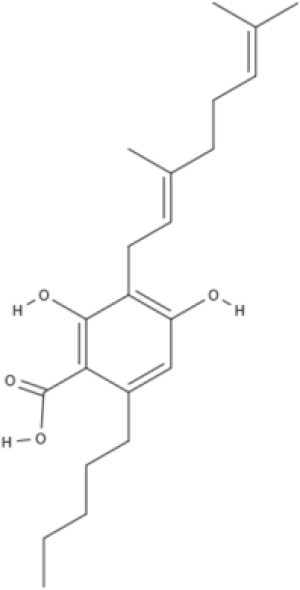	CBC 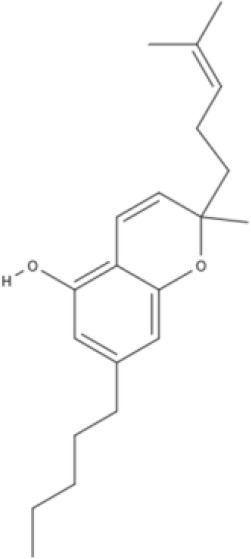
**Synthetic cannabinoids**			
**Dibenzopyran derivatives (THC related compounds)**			**Cyclohexylphenol derivatives**
HU-210 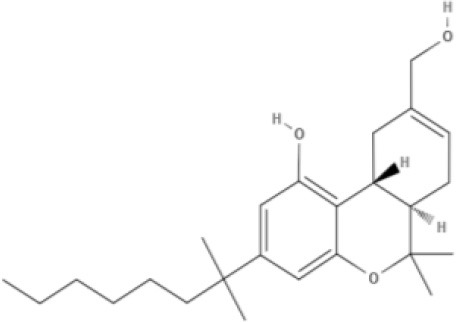		Ajulemic acid 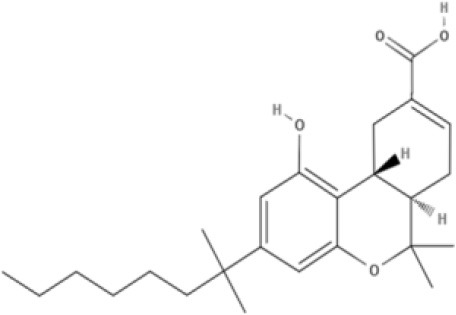	CP55940 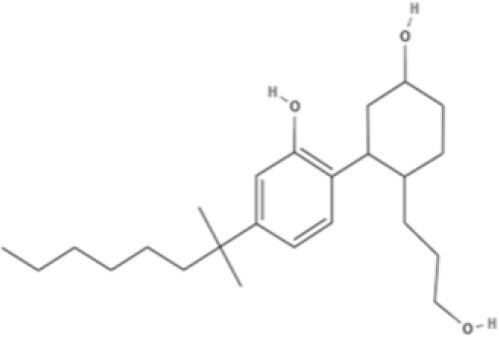
**Aminoalkylindoles**			
JWH-015 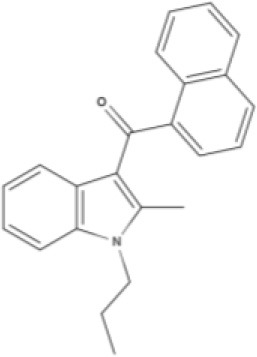	WIN 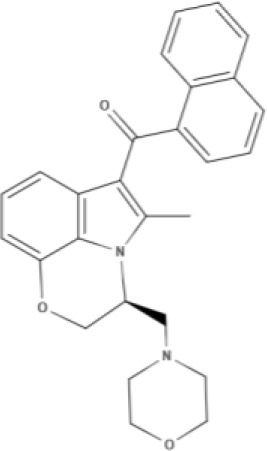	AM630 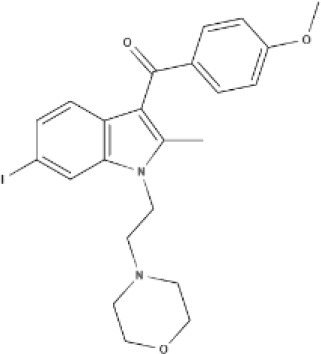	
**Synthetic analogs of ECs**			
MA 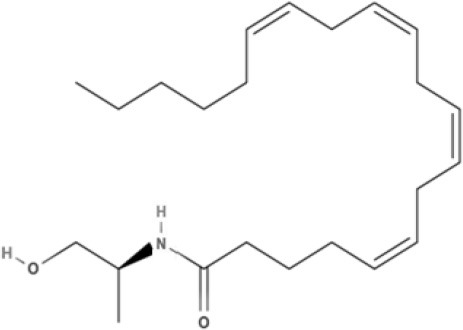	ACPA 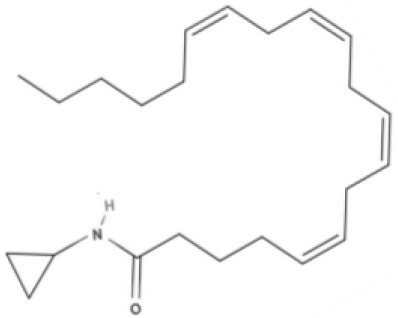		
**Other synthetic cannabinoids**			
O-1918 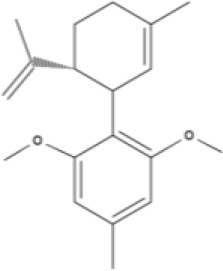	O-1602 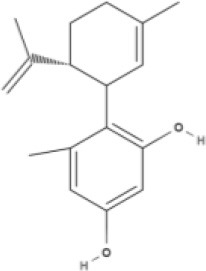	HU-308 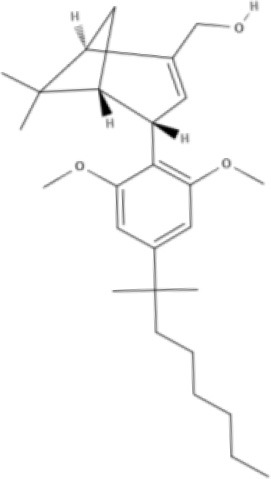	LY320135 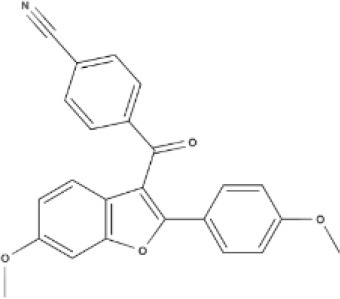
SR141716A 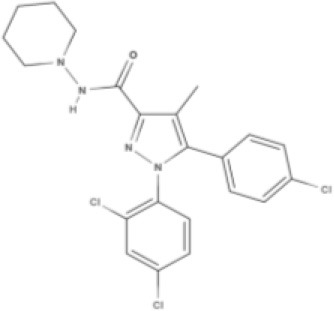	AM251 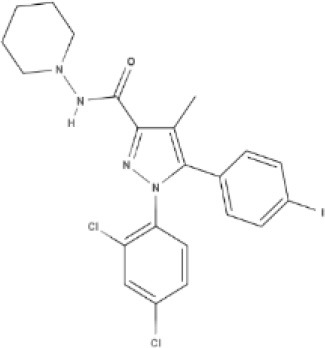		

*AEA, arachidonoyl ethanolamide or anandamide; 2-AG, 2-arachidonoyl glycerol; OEA, oleoylethanolamine; PEA, palmitoylethanolamide; NAGly, N-arachidonoyl glycine; LPI, lysophosphatidylinositol; 2A-LPA, 2-arachidonoyl lysophosphatidic acid; THC, Δ9-tetrahydrocannabinol; THCA, THC acid; THCVA, tetrahydrocannabivarin acid; CBD, cannabidiol; CBDA, cannabidiol acid; CBDV, cannabidivarin; CBDVA, cannabidivarin acid; CBN, cannabinol; CBC, cannabichromene; CBG, cannabigerol; CBGV, cannabigerovarin; CBGA, cannabigerol acid; AbCBD, abnormal cannabidiol; WIN, WIN55212-2; MA, methanandamide; ACPA, arachidonylcyclopropylamide*.

It is now clear that, in addition to the classic CB1 and CB2 receptors, cannabinoid-related agents interact with a spectrum of macromolecular targets, including: other receptors; ion channels; transporters; enzymes and; protein- and non-protein cellular structures. Evidence for cannabinoid modulation of these targets has already been the subject of excellent reviews (Kreitzer and Stella, 2009; De Petrocellis and Di Marzo, 2010; Pertwee, 2010). The purpose of this review is to examine current studies that focus on how off-receptor targets mediate the effects of classical cannabinoids in the CNS and in cancer. Cannabinoids that interact with cannabinoid receptors in an allosteric manner (allosteric modulators) are outside of the scope of this review but have been recently reviewed elsewhere (Busquets Garcia et al., 2016; Janero and Thakur, 2016; Khurana et al., 2017; Nguyen et al., 2017).

Active constituents of *Cannabis* produce CNS effects, and the endocannabinoid signaling system was discovered in CNS, therefore cannabinoid effects on neuronal activity have been more thoroughly and efficiently studied than in more peripheral processes like cancer. Although CB1 and/or CB2 receptors are known to be expressed in some cancers (Sarfaraz et al., 2008), their levels vary and may be either up- or down regulated (Bifulco et al., 2001; Begum et al., 2005). Variable receptor expression suggests that cannabinoid effects in cancer are more likely to involve non-receptor mechanisms than in CNS, making the system a promising area to examine for novel targets. Thus, focusing on both CNS and cancer will allow established central and emerging peripheral effects that are not mediated by CB1 or CB2 to be compared and contrasted, in order to appreciate common and divergent mechanisms that are involved. This comparison may also reveal important cannabinoid targets in the CNS and cancer that can form a basis for inquiry in other organ systems. In addition, awareness of non-cannabinoid receptor targets of cannabinoids may lead to the development of drugs with greater efficacy and specificity for their targets.

### Roles of cannabinoids and the cannabinoid receptors in the CNS

CB1 receptors are abundant in CNS and expressed in brain at consistently high densities across vertebrate species within which levels have been measured (~2,000 fmol/mg protein, e.g., Pertwee, 1997; Soderstrom et al., 2000; Soderstrom and Johnson, 2001). Although expressed at much lower levels than CB1, CB2 receptors also play clear roles in reward-related CNS activity (Zhang et al., 2014) and immune responses (Cabral et al., 2008). High-level CB1 expression (approaching that of amino acid transmitter receptors) may have contributed to delayed appreciation of central cannabinoid effects produced following interaction with other cellular macromolecules. This is because the magnitude of non-receptor-mediated effects may be small relative to those that follow activation of the more abundant CB1 receptors. This is of therapeutic importance as when using drugs to treat disease often “less is more” and more modest indirect effects may be adequate to mitigate disease processes while avoiding toxicity. Thus, cannabinoid-related agents with targets independent of CB1 and CB2 receptors are currently being effectively employed or evaluated for management of a variety of CNS disorders (McPartland et al., 2014). The non-CB1/CB2 CNS-relevant targets reviewed here, along with the cannabinoid ligands that interact with them, are summarized in Table 2.

**Table 2:** Summary of CNS-relevant non-cannabinoid receptor targets.

**Target**	**Target type**	**Ligand**	**Ligand type**	**Activity**	**Potency (EC50, nM)**	**System**	**Assay**	**References**
5-HT1A	Receptor	CBD	Phyto	Agon	NA	Rat BNST	Anxiolysis	Gomes et al., 2011
5-HT1A	Receptor	CBD	Phyto	Agon	~3,000	LN 231	Cell viab.	Ward et al., 2014
5-HT1A	Receptor	CBD	Phyto	Agon	NA	CHO	Cyclase inhib.	Russo et al., 2005
5-HT1A	Receptor	CBD	Phyto	Agon	~1 μg/kg	Rat	Gaping	Rock et al., 2014
5-HT1A	Receptor	CBD	Phyto	Agon	NA	CHO	GTPγS	Russo et al., 2005
5-HT1A	Receptor	CBD	Phyto	Agon	NA	Mouse	Panic	Twardowschy et al., 2013
5-HT1A	Receptor	CBD	Phyto	Agon	NA	Rat MFB	Plus maze	Fogaça et al., 2014
5-HT1A	Receptor	CBD	Phyto	Antag	NA	Rat	ICSS	Katsidoni et al., 2013
5-HT1A	Receptor	CBD	Phyto	Ligand	NA	CHO	Lig. displ.	Russo et al., 2005
5-HT3	Receptor	AEA	Endo	Antag	190	Rat NDG	Cat. current	Fan, 1995
5-HT3	Receptor	AEA	Endo	NCAnt	129	Rat NDG	Cat. current	Barann et al., 2002
5-HT3	Receptor	CP55940	Syn	Antag	94	Rat NDG	Cat. current	Fan, 1995
5-HT3	Receptor	CP55940	Syn	NCAnt	648	Rat NDG	Cat. current	Barann et al., 2002
5-HT3	Receptor	JWH-015	Syn	NCAnt	147	Rat NDG	Cat. current	Barann et al., 2002
5-HT3	Receptor	LY320135	Syn	NCAnt	523	Rat NDG	Cat. current	Barann et al., 2002
5-HT3	Receptor	THC	Phyto	NCAnt	38.4	Rat NDG	Cat. current	Barann et al., 2002
5-HT3	Receptor	WIN	Syn	Antag	310	Rat NDG	Cat. current	Fan, 1995
5-HT3	Receptor	WIN	Syn	NCAnt	104	Rat NDG	Cat. current	Barann et al., 2002
A_2A_	Receptor	CBD	Phyto	Agon	NA	EOC-20	Cell prolif.	Carrier et al., 2006
AMPA	Receptor	AEA	Endo	Antag	160–240	X. oocytes	Cat. current	Akinshola et al., 1999
ANA trans	Transporter	CBD	Phyto	Antag	25,300	leukemia Cx	ANA uptake	De Petrocellis et al., 2011
ANA trans	Transporter	CBG	Phyto	Antag	11,300	leukemia Cx	ANA uptake	De Petrocellis et al., 2011
DAGLα	Enzyme	THCA	Phyto	Antag	27,300	COS-7	2-AG met	De Petrocellis et al., 2011
δOP	Receptor	SR141716A	Syn	NCAnt	NA	CB1 KO	GTPγS	Zádor et al., 2014
ENT1	Transporter	CBD	Phyto	Antag	~250	EOC-20	Nucleoside	Carrier et al., 2006
ENT1	Transporter	THC	Phyto	Antag	~50	EOC-20	Nucleoside	Carrier et al., 2006
FAAH	Enzyme	CBD	Phyto	Antag	15,200	Rat brain	ANA met	De Petrocellis et al., 2011
GABA_A_ B_2_	Receptor	2-AG	Endo	Agon	1,100	X. oocytes	Cl^−^ current	Sigel et al., 2011
GABA_A_ B_2_	Receptor	2-AG	Endo	Antag	NA	HEK293	Cl^−^ current	Golovko et al., 2014
GABA_A_ B_2_	Receptor	CP55940	Syn	Antag	NA	HEK293	Cl^−^ current	Golovko et al., 2014
Glycine	Receptor	2-AG	Endo	Antag	NA	HCX neurons	Cl^−^ current	Lozovaya et al., 2005
Glycine	Receptor	AEA	Endo	Agon	230–318	Rat VTA	Cl^−^ current	Hejazi et al., 2006
Glycine	Receptor	AEA	Endo	Antag	300	HCX neurons	Cl^−^ current	Lozovaya et al., 2005
Glycine	Receptor	THC	Phyto	Agon	115	Rat VTA	Cl^−^ current	Hejazi et al., 2006
Glycine	Receptor	WIN	Syn	Antag	300	HCX neurons	Cl^−^ current	Lozovaya et al., 2005
Glycine α1	Receptor	AEA	Endo	Antag	38	HEK293	Cl^−^ current	Yang et al., 2008
Glycine α2	Receptor	HU210	Syn	Antag	90	HEK293	Cl^−^ current	Yang et al., 2008
Glycine α2	Receptor	HU308	Syn	Antag	1,130	HEK293	Cl^−^ current	Yang et al., 2008
Glycine α2	Receptor	NAGly	Endo	Antag	3,030	HEK293	Cl^−^ current	Yang et al., 2008
Glycine α2	Receptor	WIN	Syn	Antag	220	HEK293	Cl^−^ current	Yang et al., 2008
GLYT1a	Transporter	AEA	Endo	Agon	30,000	X. oocytes	Gly trans	Pearlman et al., 2003
GPR119	Receptor	OEA	Endo	Agon	3,200	Yeast	LacZ	Overton et al., 2006
GPR119	Receptor	PEA	Endo	Agon	>10,000	Yeast	LacZ	Overton et al., 2006
GPR18	Receptor	AbCBD	Syn	Agon	~0.3 μg/kg	RVLM	BP	Penumarti and Abdel-Rahman, 2014b
GPR18	Receptor	AbCBD	Syn	Agon	836	HEC-1B	MAPK act	McHugh et al., 2012
GPR18	Receptor	ACPA	Syn	Agon	1,350	HEC-1B	MAPK act	McHugh et al., 2012
GPR18	Receptor	AM251	Syn	Antag	9,640	HEC-1B	MAPK act	McHugh et al., 2012
GPR18	Receptor	AEA	Endo	Agon	383	HEC-1B	MAPK act	McHugh et al., 2012
GPR18	Receptor	CBD	Phyto	Antag	5,110	HEC-1B	MAPK act	McHugh et al., 2012
GPR18	Receptor	NAGly	Endo	Agon	NA	CHO	Ca^++^	Kohno et al., 2006
GPR18	Receptor	NAGly	Endo	Agon	~30	CHO	Cyclase inhib.	Kohno et al., 2006
GPR18	Receptor	NAGly	Endo	Agon	45	HEC-1B	MAPK act	McHugh et al., 2012
GPR18	Receptor	O-1602	Syn	Agon	65	HEC-1B	MAPK act	McHugh et al., 2012
GPR18	Receptor	O-1918	Syn	Antag	~0.4 μg/kg	RVLM	BP	Penumarti and Abdel-Rahman, 2014b
GPR18	Receptor	THC	Phyto	Agon	960	HEC-1B	MAPK act	McHugh et al., 2012
GPR35	Receptor	2A-LPA	Endo	Agon	~100	HEK293	Ca++	Oka et al., 2010
GPR55	Receptor	2-AG	Endo	Agon	3	HEK293	GTPγS	Ryberg et al., 2007
GPR55	Receptor	AbCBD	Phyto	Agon	2,500	HEK293	GTPγS	Ryberg et al., 2007
GPR55	Receptor	AM251	Syn	Agon	39	HEK293	GTPγS	Ryberg et al., 2007
GPR55	Receptor	AEA	Endo	Agon	18	HEK293	GTPγS	Ryberg et al., 2007
GPR55	Receptor	CBD	Phyto	Antag	NA	HEK293	GTPγS	Ryberg et al., 2007
GPR55	Receptor	CP55940	Syn	Agon	5	HEK293	GTPγS	Ryberg et al., 2007
GPR55	Receptor	HU210	Syn	Agon	26	HEK293	GTPγS	Ryberg et al., 2007
GPR55	Receptor	LPI	Endo	Agon	200	HEK293	ERK	Oka et al., 2007
GPR55	Receptor	Noladin Ether	Endo	Agon	10	HEK293	GTPγS	Ryberg et al., 2007
GPR55	Receptor	O-1602	Syn	Agon	13	HEK293	GTPγS	Ryberg et al., 2007
GPR55	Receptor	OEA	Endo	Agon	440	HEK293	GTPγS	Ryberg et al., 2007
GPR55	Receptor	PEA	Endo	Agon	4	HEK293	GTPγS	Ryberg et al., 2007
GPR55	Receptor	THC	Phyto	Agon	8	HEK293	GTPγS	Ryberg et al., 2007
GPR55	Receptor	Virodhamine	Endo	Agon	12	HEK293	GTPγS	Ryberg et al., 2007
κOP	Receptor	SR141716A	Syn	InvAg	NA	CB1 KO	GTPγS	Zádor et al., 2015
MAGL	Enzyme	CBG	Phyto	Antag	95,700	COS	2-AG met	De Petrocellis et al., 2011
MAGL	Enzyme	THCA	Phyto	Antag	46,000	COS	2-AG met	De Petrocellis et al., 2011
μOP	Receptor	Noladin Ether	Endo	NCAnt	NA	CB1 KO	GTPγS	Zádor et al., 2012
μOP	Receptor	SR141716A	Syn	Antag	NA	CB1 KO	GTPγS	Cinar and Szücs, 2009
μOP	Receptor	THC	Phyto	Agon	NA	Rat *in vivo*	Hot plate	Tulunay et al., 1981
Na^+^ channel	Ion Channel	Ajulemic acid	Syn	Antag	~3,000	HEK293	Na^+^ current	Foadi et al., 2014
nAChR a4β2	Receptor	AEA	Endo	Antag	NA	SH-EP1	Na^+^ current	Spivak et al., 2007
nAChR α7	Receptor	2-AG	Endo	Antag	118	X. oocytes	Na^+^ current	Oz et al., 2004
nAChR α7	Receptor	AEA	Endo	Antag	30	X. oocytes	Na^+^ current	Oz et al., 2003
nAChR α7	Receptor	CBD	Phyto	Antag	11,300	X. oocytes	Na^+^ current	Mahgoub et al., 2013
nAChR α7	Receptor	MA	Syn	Antag	~1 μmol/kg	Anesth. Rat	HR	Baranowska et al., 2008
NCX1	Transporter	AEA	Endo	Antag	4,700	Rat myocytes	Na^+^/Ca^++^	Kury et al., 2014
NMDA	Receptor	AEA	Endo	Agon	NA	Rat ICV	BP	Malinowska et al., 2010
NMDA	Receptor	AEA	Endo	Agon	NA	Rat HCX	Ca^++^ current	Yang et al., 2014
TRPA1	Ion Channel	AM251	Syn	Agon	10,000	CHO	[Ca^++^]i	Patil et al., 2011
TRPA1	Ion Channel	CBD	Phyto	Agon	110	HEK293	[Ca^++^]i	De Petrocellis et al., 2011
TRPA1	Ion Channel	CBG	Phyto	Agon	700	HEK293	[Ca^++^]i	De Petrocellis et al., 2011
TRPA1	Ion Channel	THC	Phyto	Agon	230	HEK293	[Ca^++^]i	De Petrocellis et al., 2011
TRPM8	Ion Channel	CBD	Phyto	Antag	60	HEK293	[Ca^++^]i	De Petrocellis et al., 2011
TRPM8	Ion Channel	CBG	Phyto	Antag	160	HEK293	[Ca^++^]i	De Petrocellis et al., 2011
TRPM8	Ion Channel	THC	Phyto	Antag	160	HEK293	[Ca^++^]i	De Petrocellis et al., 2011
TRPV1	Ion Channel	AM251	Syn	Agon	10,000	CHO	[Ca^++^]i	Patil et al., 2011
TRPV1	Ion Channel	AM630	Syn	Agon	10,000	CHO	[Ca^++^]i	Patil et al., 2011
TRPV1	Ion Channel	CBD	Phyto	Agon	1,000	HEK293	[Ca^++^]i	De Petrocellis et al., 2011
TRPV1	Ion Channel	CBG	Phyto	Agon	1,300	HEK293	[Ca^++^]i	De Petrocellis et al., 2011
TRPV2	Ion Channel	CBD	Phyto	Agon	1,250	HEK293	[Ca^++^]i	De Petrocellis et al., 2011
TRPV2	Ion Channel	THC	Phyto	Agon	650	HEK293	[Ca^++^]i	De Petrocellis et al., 2011

*ICSS, intracranial self stimulation; HR, heart rate; BP, blood pressure; MAPK act, MAPK activation; Cat. current, Cation current; AEA, arachidonoyl ethanolamide or anandamide; MA, methanandamide; CBD, cannabidiol; CBG, cannabigerol; Agon, agonist; NCAnt, non-competitive antagonist; Antag, antagonist; NDG, nodose ganglion; Cx, culture; Lig. displ., displacement of ligand binding; MFB, medial forebran bundle*.

### Effects of cannabinoids and the cannabinoid receptors on cancer

Both CB1 and CB2 receptors, and their endogenous ligands, are expressed in a variety of peripheral organs, including; the GI tract, liver, bone, reproductive system, skin, and the immune system. Peripheral endocannabinoid signaling also regulates many aspects of human pathophysiology, including cancer. Cannabinoid agonists are used as palliative therapy for chemotherapy-induced nausea and vomiting, and they may also be beneficial in the treatment of cancer-related pain. Evidence from both *in vitro* and *in vivo* studies established the efficacy of these compounds in reducing tumor growth and proliferation (Ladin et al., 2016). Much of this efficacy is attributable to interaction with targets other than the classic CB1/CB2 receptors. Therefore, we focus on how off-receptor targets mediate cannabinoid effects on cancer. The cancer-related off-receptor targets reviewed here, and the cannabinoid ligands that interact with them, are summarized in Table 3.

**Table 3:** Summary of cancer-relevant non-cannabinoid receptor targets.

**Cancer type**	**Cell line**	**Drug**	**Target**	**Activity**	**Cellular response**	**Assay**	**Reference**
Colon and colorectal cancer	HT29	AEA	FAAH	Substrate	Reduced cell death	Adherent cell count	Patsos et al., 2005
	HCA7	AEA	COX-2	Substrate	Cell death	Adherent cell count	Patsos et al., 2005
	HCT116 Caco-2	CBG	TRPM8	Antagonist	Apoptosis	• Caspase 3/7 activity • DNA fragmentation	Borrelli et al., 2014
	SW480 HT29	WIN	Phosphatase	Increased expression, Activation	Apoptosis	• PARP cleavage • TUNEL	Sreevalsan and Safe, 2013
	SW480	WIN	Phosphatase	Increased expression	Reduced proliferation, Apoptosis	• Cell number • PARP cleavage • Caspase 3	Sreevalsan et al., 2011
Brain tumor:							
Glioma	Ge227 Ge258 U87 U251	AEA	TRPV1	Agonist	Apoptosis	DNA fragmentation	Contassot et al., 2004b
	U87	CBD	TRPV2	Agonist, Increased expression	Increased chemotherapeutic sensitivity, Inhibition of cell migration	• MTT (cell viability) • Chemotaxis (cell migration)	Nabissi et al., 2013
	H4	MA	COX-2	Increased expression	Apoptosis	• Caspase • PARP cleavage	Eichele et al., 2006
	GSC patient derived	CBD	TRPV2	Agonist, Increased expression	Increased differentiation, Autophagy, Reduced proliferation	• Flow (differentiation) • LC3I, LC3II (autophagy) • MTT (proliferation)	Nabissi et al., 2015
Neuroblastoma	N1EE-115	AEA	FAAH	Substrate	Reduced cell death	• MTT	Hamtiaux et al., 2011
Prostate	LNCaP	WIN	Phosphatase	Increased expression	Reduced proliferation, Apoptosis	• Cell number • PARP cleavage • Caspase 3	Sreevalsan et al., 2011
Non-melanoma skin cancer	JWF2	AEA	COX-2	Substrate	Apoptosis	• PARP cleavage • Caspase 3	Kuc et al., 2012
	JWF2	AEA	FAAH	Substrate	Reduced apoptosis	• PARP cleavage • Caspase 3 • TUNEL	Kuc et al., 2012
Lung cancer	A549 H460	CBD	PPAR⋎ COX-2	Activation of PPAR⋎ & COX-2, Increased COX-2 expression	Apoptosis, Reduced tumor growth (xenograft)	DNA fragmentation	Ramer et al., 2013
Murine lung cancer	L1C2	MA	COX-2	Expression and activity	Increased tumor growth	Tumor volume	Gardner et al., 2003
	HeLa	MA	PPAR⋎ COX-2	Activation of PPAR⋎ & COX-2, Increased COX-2 expression	Apoptosis	DNA fragmentation	Eichele et al., 2009
Cervical cancer	HeLa Caski CC299	AEA	TRPV1	Agonist	Apoptosis	• subG0/G1 • Caspase 7 cleavage	Contassot et al., 2004a
Cholangiocarcinoma	Mc-ChA-1	AEA	GPR55	Activation	Apoptosis	Annexin V	DeMorrow et al., 2007
Multiple myeloma	RPMI, U266	CBD	TRPV2	Agonist	Necrosis, Cell death, Increased therapeutic sensitivity	• Propidium iodide (necrosis) • MTT (cell death)	Morelli et al., 2014

*AEA, arachidonoyl ethanolamide or anandamide; FAAH, fatty acid amide hydrolase; CBG, cannabigerol; TRPM8, transient receptor potential cation channel subfamily M member 8; WIN, WIN55212-2; CBD, cannabidiol; MA, methanandamide; PPAR⋎, peroxisome proliferator-activated receptor gamma; COX-2, cyclooxygenase-2; TRPV1, TRPV2, transient receptor potential cation channel subfamily V members 1 and 2; GPR55, G protein-coupled receptor 55; MTT, 3-(4,5-dimethythiazol-2-yl)-2,5-diphenyl tetrazolium bromide; PARP, Poly (ADP-ribose) polymerase; TUNEL, terminal deoxynucleotidyl transferase dUTP nick end labeling*.

## Non-CB1/CB2 receptor targets

### Deorphanized G-protein-coupled receptors (GPCRs) in CNS and cancer

Four former orphan GPCRs (GPR55, GPR18, GPR119, and GPR35) have now been clearly established to transduce effects of a subset of both naturally-occurring and synthetic cannabinoid compounds. The available data demonstrate that cannabinoids directly activate these four GPCRs in the CNS, and they also activate GPR55 in cancer.

#### GPR55 in CNS

The most well-characterized of the deorphanized GPCRs, GPR55 (Ryberg et al., 2007), is expressed most highly in brain, adrenal gland, and digestive tract (although at reportedly low levels relative to that of CB1). When expressed in HEK293 cells, specific binding of the synthetic CB1 agonist CP55940 is observed, but not of the CB1/CB2 synthetic agonist WIN55212-2. Interestingly, a very small amount of specific GPR55 binding was observed using 50 nM of the CB1-selective antagonist/inverse agonist SR141716A (SR).

Using the same HEK293 cell line for GTPγS functional assays, the endogenous agonist 2-arachidonylglyerol (2-AG) potently activates GPR55 (EC50 = 3 nM), although virodhamine (EC50 = 12 nM) is a more complete agonist with about 50% greater efficacy. Notably, in a separate assay of intracellular calcium release, 2-AG and virodhamine showed no GPR55 agonism, suggesting this receptor is subject to agonist-dependent functional selectivity (Lauckner et al., 2008). Other potent agonists discovered in this initial screen of GPR55 efficacy include; palmitoylethanolamide (PEA), CP55940, Δ^9^-tetrahydrocannabinol (THC), noladin ether, and anandamide with EC50s of 4, 5, 8, 10, and 18 nM respectively, and efficacies similar to that of 2-AG. Interestingly, the non-psychoactive phytocannabinoid cannabidiol (CBD) was found to antagonize GPR55 activity at physiologically relevant concentrations.

GPR55 primarily couples through the relatively obscure Gα13. This interesting G-protein activates a cascade involving RhoA that, among other effects, ultimately alters actin polymerization and stability, implicating GPR55 signaling in processes related to neuronal morphology (Worzfeld et al., 2008). This potential role is further supported by effects to promote neurite retraction (Obara et al., 2011). More recently, evidence in transfected HEK293 cells indicates that signaling through G_q_ also occurs (Lauckner et al., 2008).

In ERK activation assays with GPR55-expressing HEK293 cells, lysophosphatidylinositol (LPI) is implicated as an important endogenous agonist (Oka et al., 2007). Notably, 2-AG and virodhamine were not effective in stimulating ERK in transfected HEK293 cells, possibly indicating ligand-dependent functional selectivity for different signal transduction pathways. LPI was also found to dose-dependently increase intracellular calcium in these cells with an EC50 ~ 1 μM, an effect possibly related to GPR55 activation of G_q_. System-dependent efficacy of GPR55 signaling may also involve interaction with other receptors including CB1. GPR55 and CB1 have been shown to heterodimerize within rat and macaque striatum, possibly related to a role in motor behaviors. When co-expressed in HEK293 cells, CB1 and GPR55 antagonize respective agonist effects to promote ERK1/2 activation (Martínez-Pinilla et al., 2014).

The physiological significance of GPR55 expression in brain remains an open question; although, some evidence indicates a role in controlling ingestive behaviors (reviewed by Liu et al., 2015). A role in promoting appetite makes sense as it has been reported that GPR55 regulates peripheral metabolism and energy homoeostasis (reviewed by Simcocks et al., 2014). Deletion of GPR55 in mice has subtle effects on motor coordination, but it has shown no significant effect on several learning and memory tests, in contrast to the role of CB1 signaling, and despite dense GPR55 expression in hippocampus, striatum, and cortex (Wu et al., 2013). Spinal cord expression of GPR55 is upregulated in a chronic constriction injury model of neuropathic pain; this suggests that it may mediate some of the analgesic effects of N-arachidonoyl-serotonin (AA-5-HT, Malek et al., 2016). GPR55 activation by some agonists increases calcium release from intraneuronal stores and inhibits M-type potassium current, both tending to promote neuronal activity (Lauckner et al., 2008). These effects may be important in the context of CBD's ability to antagonize the receptor, as this non-psychoactive phytocannabinoid has recently been shown effective for the treatment of Dravet syndrome, a formerly intractable form of childhood epilepsy (Devinsky et al., 2017). Whether the efficacy of CBD in this syndrome involves GPR55 antagonism, or perhaps, involves some combination of the many other established targets of this drug (see Table 2) remains to be resolved. Other evidence for potential roles for GPR55 signaling includes: distinct expression in rod cells of primate retina suggesting a role in low-light vision (Bouskila et al., 2013) and; expression in microglia that, following LPI activation, acts to protect hippocampal neurons from excitotoxicity (Kallendrusch et al., 2013).

#### GPR55 in cancer

Despite the numerous studies illuminating the physiological functions of GPR55 in the CNS described above (section GPR55 in CNS), limited information is available about the role of this receptor in cancer. GPR55 is expressed in different cancer cell types raising the possibility that it may be a target for cancer chemotherapeutic agent development (reviewed by Falasca and Ferro, 2016). Much of the research conducted thus far indicates that GPR55 promotes tumor formation. GPR55 expression was examined in human tumor biopsy samples from breast, pancreatic, and glioblastoma patients (Andradas et al., 2011). In this study, high levels of GPR55 were strongly correlated with the aggressiveness of the malignancy. Additional evidence for GPR55-promotion of malignancy includes; (1) elevated levels of its endogenous ligand lysophosphatidylinositol (LPI) in ovarian cancers (Xiao et al., 2001) and; (2) exogenous LPI promotion of proliferation and migration of various cancer cell lines (Ford et al., 2010; Piñeiro et al., 2011). However, when GPR55 is activated by the endocannabinoid, AEA, cholangiocarcinoma cell survival is inhibited. AEA induces cholangiocarcinoma cell apoptosis by recruiting Fas death receptors into lipid rafts and activating the JNK signaling pathway (DeMorrow et al., 2007; Huang et al., 2011). Genetic disruption of GPR55 receptor expression using shRNA blocked the antiproliferative effects of AEA *in vitro* and *in vivo* (Huang et al., 2011). Of note, AEA-induced cell death was not reversed in the presence of CB1- (SR141716A) or CB2- (SR144528) selective antagonists although each cannabinoid receptor was expressed (DeMorrow et al., 2007). These findings suggest that AEA elicits antiproliferative effects on cholangiocarcinoma that are GPR55-dependent and CB receptor-independent. Other deorphanized GPCRs, including GPR18 and GPR35, have been identified as modulators of tumorigenesis (Okumura et al., 2004; Qin et al., 2011); however, the role of cannabinoid ligands in these systems is unclear. Examination of the deorphanized cannabinoid receptors in cancer will provide greater insight into their impact on tumor development and progression. In addition, determining whether classical cannabinoids mediate their effects through the deorphanized receptors will shed light on mechanisms of their antitumor activity.

#### GPR18 in CNS

Interestingly, microglia migration is stimulated by activation of a second formerly orphan cannabinoid-related receptor, GPR18 (McHugh et al., 2010). This G_i/o_-coupled receptor was originally reported to be highly-expressed in testes and spleen (Gantz et al., 1997); yet, it clearly is also functional within brainstem (Penumarti and Abdel-Rahman, 2014b). GPR18 is activated by N-arachidonoyl glycine (NAGly, Kohno et al., 2006) that is a product of anandamide metabolism (Aneetha et al., 2009; Bradshaw et al., 2009). It is also activated by abnormal cannabidiol (abn-CBD) that, when infused into regions of the brainstem, causes reduction in blood pressure in a manner that involves nitric oxide synthase and adiponectin signaling (Penumarti and Abdel-Rahman, 2014a). As with GPR55 discussed above (section GPR55 in CNS), GPR18 receptor expression is upregulated following spinal cord injury; this suggests that it may mediate some of the analgesic effects of N-arachidonoyl-serotonin (AA-5-HT, Malek et al., 2016). Notably, the principal active constituent of *Cannabis*, THC, is a potent agonist of GPR18 when expressed in HEK293 cells (McHugh et al., 2012). The lesser *Cannabis* constituent, CBD, along with synthetic CB agonists CP55940 and WIN55212-2, have no activity at physiologically-relevant concentrations. Potent GPR18 activation by THC and NAGly has been suggested to underlie efficacy to resolve inflammatory pain (Burstein et al., 2011; McHugh et al., 2014; Crowe et al., 2015). Some controversy about GPR18 signal transduction was raised when NAGly failed to inhibit Ca^++^ currents following heterologous expression and agonist stimulation in rat superior cervical ganglion neurons (Lu et al., 2013). Whether this is a special case of system-dependent functional selectivity, or an indication that NAGly modulates neuronal activity via other receptor systems, remains an open question.

#### GPR119 in CNS

A third relevant G-protein-coupled receptor, GPR119, has been deorphanized. In humans, GPR119 was initially reported to be expressed only outside of CNS, most notably in pancreas, suggesting a peripheral metabolic function (Fredriksson et al., 2003). A possible CNS expression difference between human and rodent species is suggested in a patent application that indicates GPR119 is also expressed at high density in rat and mouse brain (Bonini et al., 2002). Mouse CNS expression is confirmed by high-affinity GPR119 binding and potent efficacy to reduce seizure-related hippocampal activity (Scott et al., 2014). At micromolar concentrations, the endogenous peroxisome proliferator-activated receptor alpha (PPARα) agonist oleoylethanolamide (OEA) activates GPR119, while anandamide appears to have partial efficacy in yeast cells expressing the receptor (Overton et al., 2006). Notably, 2-AG, CP55940, WIN55212-2, methanandamide, and JWH-133 did not activate GPR119. Supporting CNS activity, and a role in controlling ingestive behaviors, OEA and a synthetic GPR119 agonist with similar efficacy, but four-fold greater potency, PSN632408, were found to reduce food consumption and body weight in Sprague-Dawley rats (Overton et al., 2006). More recent evidence suggests that PSN632408 may lack selectivity for GPR119 in peripheral tissues (Ning et al., 2008). Despite evidence of GPR119 CNS activity, this receptor is being most closely studied in as a target for treating diabetes and other metabolic disorders (Ansarullah et al., 2013; Cornall et al., 2013, 2015).

#### GPR35 in CNS

A final G_i/o_-coupled deorphanized receptor, GPR35, has been reported to transduce effects of 2-arachidonyl lysophosphatidic acid (2A-LPA) and kynurenic acid, both of which are present at relevant concentrations within neuronal tissues. 2A-LPA is a product of metabolism of the principal brain endocannabinoid, 2-arachidonyl glycerol (2-AG, Oka et al., 2010). Kynurenic acid is the product of tryptophan metabolism, and is most well-characterized as an endogenous antagonist of receptors for the principal excitatory neurotransmitter, NMDA (Stone et al., 2013). A very recent structure-activity study has led to identification of several potent GPR35 agonists and three structures critical for efficacy (Abdalhameed et al., 2017). GPR35 is expressed most predominantly in the gastrointestinal tract and in leukocytes (Wang et al., 2006). Low-level expression of GPR35 in brain reduces the likelihood that it significantly modulates neuronal activity within this region of the CNS. Within spinal cord, on the other hand, convincing evidence demonstrates functionally-relevant expression of GPR35, with distinct enrichment within murine dorsal horn ganglia (Cosi et al., 2011). In an acetic acid-induced pain assay, both kynurenic acid, and the phosphodiesterase inhibitor, zaprinast, were found to produce significant analgesia with EC50s of about 100 and 1 mg/kg, respectively. Note zaprinast is an effective agonist of GPR35 with a particularly high potency at rat vs. human forms of the receptor (Taniguchi et al., 2006). Homology between GPR35, and other LPA receptors, may suggest that 2A-LPA is more relevant than kynurenic acid as an endogenous modulator (Zhao and Abood, 2013). Clearly, more needs to be learned and understood about GPR35 signaling.

### Opioid receptors in CNS

Evidence implicating interaction between cannabinoid and opioid signaling systems began to accumulate early, and before specific cannabinoid receptors were unequivocally identified. For example, it was well-established by the 1980s that THC effectively reduces symptoms of naloxone-precipitated opioid withdrawal (Hine et al., 1975). Similarly, the analgesic efficacy of THC was known to depend upon μ-opioid receptors (μOP, Cox et al., 2015), as use of an irreversible antagonist (chlornaltrexamine, that alkylates and destroys activity of μOPs) reduces efficacy of both morphine and THC in the hotplate assay (Tulunay et al., 1981). Using the μOP-selective radioligand, [^3^H]-dihydromorphine, it was found that THC non-competitively reduces the density of binding sites in brain membranes via an unknown mechanism (Vaysse et al., 1987). More recently, it has been demonstrated that augmentation of endocannabinoid signaling using the monoacylglycerol lipase (MAGL) inhibitor JZL184 (MAGL is an enzyme responsible for degrading the endocannabinoid 2-AG) has efficacy similar to THC in reducing opioid withdrawal symptoms. This suggests MAGL as a potential target for managing opioid addiction (Ramesh et al., 2011).

More progress has been made since development of CB1/CB2 receptor-deficient mice and availability of selective antagonists. Interestingly, when using CB1 knockout mice with the CB1-selective cannabinoid antagonist/inverse agonist SR, it was found that SR effectively reduces both basal levels of G-protein activation and the ability of the μOP peptide agonist, DAMGO, to stimulate GTPγS binding (Cinar and Szücs, 2009). These results were obtained using mouse cortical membranes (where μOP and CB1 are normally both distinctly expressed), and there was no difference in efficacy across tissue obtained from wild type and CB1 knockout animals, clearly demonstrating a non-CB1-related mechanism for SR's inverse agonist activity (although not excluding the possibility that CB1 constitutive activity drives inverse agonism in other systems). Additional experiments using CB1 and μOP transfected CHO cells revealed that micromolar concentrations of SR are able to displace ligands bound to μOPs, demonstrating an interesting direct SR-μOP interaction.

The nature of this SR-μOP interaction was further explored by demonstrating that both SR and the 2-AG-related endocannabinoid noladin ether are both able to potently antagonize DAMGO-stimulated GTPγS binding in membranes taken from wildtype animals (Zádor et al., 2012). In membranes from CB1 deficient mice, both SR and a combination of SR and noladin ether reduced the efficacy of DAMGO to stimulate GTPγS binding, without altering potency, suggesting μOP-CB1 interaction, and non-competitive antagonism of μOP in the absence of CB1. These studies employing DAMGO, relevant to μOP activity, were extended to employ the δ-opioid receptor (δOP) selective peptide agonist DPDPE. In CHO cells expressing δOP, SR reduced basal GTPγS activation with an EC50 ~ 1 μM. SR also non-competitively antagonized DPDPE stimulation of GTPγS binding to an extent similar to that achieved by the competitive δOP antagonist, naltrindole (Zádor et al., 2014). Completing evaluation of SR interaction with the three primary opioid receptor subtypes, the same group demonstrated SR displaces binding of the κ-opioid receptor (κOP) agonist U-69,593 from membranes prepared from transfected CHO cell membranes. Using these transfected cells *in vitro*, SR was found to inhibit basal levels of κOP activation in a manner antagonized by the κ-selective antagonist, nor-BNI, suggesting that SR is a κOP inverse agonist. In both CB1 deficient and wildtype mice, systemic pretreatment of animals with a low, 0.1 mg/kg dosage of SR decreased efficacy of the κOP peptide agonist, dynorphin A to activate GTPγS binding in preparations of brain membranes (Zádor et al., 2015). The magnitude of efficacy reductions following SR pretreatment was similar across CB1 knockouts and wildtype animals, and the potency was not altered, suggesting that SR acts to effectively reduce κOP density. Behavioral tests demonstrated an anxiolytic effect of low-dose (0.1 mg/kg subcutaneous) SR treatments, perhaps contrasting with established dysphoric effects of higher doses in other systems, including humans.

Related to morphine dependence, is evidence indicating that chronic morphine treatment of rats results in upregulation of both CB1 protein and mRNA encoding the receptor (Jin et al., 2014). Morphine-induced CB1 expression was also associated with cytokine release, including IL-6, and, most notably, IL-1B, in various brain regions, including cortex, hippocampus, cerebellum, and brain stem. Coincidently, CB1 upregulation and cytokine release implicate both cannabinoid signaling and immune responsiveness in effects associated with opioid dependence. Interestingly, the microRNA let-7d and CB1 receptors reciprocally downregulate each other in transfected SH-SY5Y cell cultures (Chiarlone et al., 2016). Let-7d-expressing SH-SY5Y cells show decreased sensitivity to methanandamide and morphine in stimulating ERK phosphorylation and a high degree of cross-tolerance following chronic treatments with the cannabinoid- and opioid agonists. Perhaps related to this interaction is evidence for efficacy of the opioid antagonist naltrexone (currently indicated for managing alcoholism) in the treatment of *Cannabis* dependence (Haney et al., 2015).

### Serotonin receptors in CNS

#### 5-HT3 ligand-gated cation channels in CNS

As with other systems, evidence for cannabinoid modulation of serotonin signaling was obtained soon after CB1 receptors were identified. This evidence began to accumulate through studies of the rat nodose ganglion which conducts afferent transmission from GI, heart, and lung. Activity in this ganglion is increased by the 5-HT3 subtype of serotonin-gated cation channels. Using the synthetic agonists CP55940 and WIN, and the most well-established endocannabinoid at the time, anandamide, it was discovered that each of these agonists non-competitively antagonizes serotonergic activation of 5-HT3 receptors at nanomolar concentrations (Fan, 1995). The inability to modulate cannabinoid effects on 5-HT3 activity with guanyl nucleotides suggested a non-G-protein dependent mechanism, and possible direct interaction with the ion channel. This finding raised speculation that analgesic and antiemetic efficacy of cannabinoid agonists may, at least in part, be attributable to 5-HT3 antagonism.

Effects in the isolated tissue preparation described above were corroborated by results of experiments using a human 5-HT3A-expressing CHO cell line (Barann et al., 2002). In patch clamp studies it was found that a series of synthetic, endogenous, and phytocannabinoid agonists potently inhibited serotonin activation of 5-HT3 cation channels. Notably, the phytocannabinoid THC was the most potent compound evaluated with EC50 ~ 40 nM. Cannabinoid antagonism was not inhibited by the CB1-selective antagonist SR. Specific binding of a 5-HT3-selective radioligand wasn't displaced by the cannabinoids employed, suggesting interaction with an allosteric site that doesn't influence serotonin affinity.

#### 5-HT1A GPCRs in CNS

The 5-HT1A subtype of serotonin receptors are notably associated with presynaptic distribution, where, similar to CB1 receptors, their activation reduces probability of neurotransmitter release. Although this type of presynaptic autoreceptor mechanism is well-characterized for 5-HT1A, the distribution of these receptors is wide, and it includes both excitatory and inhibitory terminals, making prediction of effects produced by modulators difficult.

Multiple lines of evidence suggest that the enigmatic non-psychotropic phytocannabinoid CBD exerts at least some of its actions through agonism of 5-HT1A autoreceptors (McPartland et al., 2015). First, CBD displaces specific binding of the 5-HT1A-selective radioligand [^3^H]8-OH-DPAT, activates GTPγS binding and inhibits adenylyl cyclase activity in heterologously expressing CHO cell cultures (Russo et al., 2005). Second, 5-HT1A receptor expression is upregulated under conditions of neuropathic pain in a manner that is reduced by cannabinoid agonism (Palazzo et al., 2006). Finally, microinfusion of CBD into the bed nucleus of the stria terminalis (BNST) dose-dependently reduces anxiety as measured by both elevated plus maze and Vogel conflict tests (Gomes et al., 2011).

These anxiolytic CBD responses are reversed by pretreatment with the 5-HT1A-selective agent WAY-100635 (WAY) that has been reported to be a neutral antagonist, although agonism at D_4_ dopamine receptors has also been reported (Chemel et al., 2006). Recently, WAY-reversible anxiolytic effects of CBD as measured in the elevated plus maze have been confirmed by others (Fogaça et al., 2014). CBD mitigation of acute restraint stress was also found, although, perhaps importantly, this effect was more resistant to WAY antagonism, possibly suggesting involvement of another target in stress vs. anxiety responses. Interestingly, anxiolytic effects of both CB1 and 5-HT1A agonists have been reported in a non-mammalian teleost fish, suggesting a conserved relationship between cannabinoid and serotonergic signaling in responding to stress (Connors et al., 2014).

Extending studies of CBD anxiolysis that are reversed by the 5-HT1A antagonist WAY, mitigation of nausea as measure in the rat gaping model (Rock et al., 2014), reduction of both neuropathic pain (Ward et al., 2014) and panicolytic effects (Twardowschy et al., 2013) have all been reported. In the context of drug abuse, CBD antagonizes effects of morphine to reduce threshold intracranial self-stimulation responding—a measure of drug reward. CBD antagonism of morphine reward was reversed by microinjection of WAY into dorsal raphe, further implicating 5-HT1A involvement (Katsidoni et al., 2013). More recently, CBD has been reported to have rapid-onset antidepressive efficacy that is dependent upon 5-HT1A activation (Linge et al., 2016).

#### Other serotonin receptors in CNS

Physiological interactions, where activity of one signaling system influences activity of another, have been documented to occur between cannabinoid and serotonergic signaling in models of chronic pain (Campos et al., 2013); epilepsy (Devinsky et al., 2014); stress-induced analgesia (Yesilyurt et al., 2015) and; tic disorders (Ceci et al., 2015). These studies reinforce the possibility of using inhibitors of endocannabinoid uptake and metabolism therapeutically.

### Adenosine receptors in CNS

Adenosine signaling in CNS is largely terminated by reuptake, making inhibitors of the transporter indirect-acting adenosinergic agonists. Such agents have shown anti-inflammatory efficacy via inhibiting release of immune mediators like TNFα (Noji et al., 2002). Interestingly, the phytocannabinoid CBD, discussed above, in the context of 5-HT1A agonism (5-HT1A GPCRs in CNS), appears to inhibit immunosuppressive effects via direct interaction with, and antagonism of, an adenosine transporter (Carrier et al., 2006). CBD anti-inflammatory effects were absent in adenosine A_2A_ knockout mice, and reversed by a selective antagonist, implicating indirect agonism of A_2A_ receptors as the mechanism of CBD-mediated immunosuppression.

Antagonists of adenosine receptors are established psychomotor stimulants. In the context of movement disorders, A_2A_ receptors are expressed at high levels within the inhibitory indirect dopaminergic pathway of vertebrate striatum. Interneurons in this region also robustly express presynaptic CB1 receptors that inhibit activity of the inhibitory pathway, resulting in an overall activation of movement-related striatal signaling. Experiments employing CB1 deficient mice found these animals to be resistant to the psychomotor stimulant effect of adenosine receptor antagonists (Lerner et al., 2010). Further investigation found that the A_2A_-selective antagonist, SCH442416, doubled the concentration of the endocannabinoid, 2-AG, within striatum, but not cortex. Increased 2-AG release was associated with decreased indirect pathway activity as measured electrophysiologically. These results demonstrate that the psychomotor efficacy of A_2A_ antagonists involve, to some extent, indirect endocannabinoid agonism. More recently it has been discovered that CB1-mediated disruption of memory consolidation is mitigated by A_2A_ antagonism, further elaborating the co-dependence of cannabinoid and adenosinergic signaling (Mouro et al., 2017).

### Excitatory amino acid receptors in CNS

#### NMDA receptors in CNS

Given general sedative effects of cannabinoid agonists, it isn't surprising that these agents act, at many sites, to reduce release of the principal excitatory neurotransmitter, NMDA. This was found to clearly be the case in rat brain slice preparations for synthetic agonists (Shen et al., 1996) and both the endocannabinoid anandamide and phytocannabinoid THC. Inhibitory effects of THC and anandamide were reversed in the presence of both the CB1-selective antagonist SR and G_i_ inhibitor pertussis toxin implicating cannabinoid-receptor involvement (Hampson et al., 1998). But, in the presence of SR, anandamide potentiation of NMDA-induced currents were noted. This stimulatory effect of anandamide was also observed in *Xenopus* oocytes expressing NMDA receptor (NMDAR) subunits, suggesting a direct agonist interaction with these cation channel proteins.

With similarities to results of experiments done in slice preparations, additional evidence for anandamide interaction with NMDA receptors derives from studies of the complex cannabinoid modulation of blood pressure control in rats (Malinowska et al., 2010). In this system, it was discovered that anandamide produces a pressor effect in the presence of the CB1-selective antagonist, SR. This increase in pressure was partially reduced by the NMDA receptor-selective antagonist MK-801, suggesting a possible direct interaction with relevance to central control of blood pressure.

More recently, the interaction of anandamide and NMDAR activation has been more elaborately studied through a series of electrophysiology studies in rat hippocampal sections and dissected cells (Yang et al., 2014). These experiments demonstrated that CA1 pyramidal cell NMDAR activation by anandamide was reversed in the presence of the TRPV1 antagonist capsazepine, raising the possibility of vanilloid receptor involvement in the anandamide effects discussed above. 2-AG was also studied in these experiments and found to produce a similar potentiation of NMDAR activation, although distinct in that it was not blocked by TRPV1 antagonism. Interestingly, the efficacy of 2-AG was increased when delivered intracellularly, suggesting interaction with NMDAR or other protein domains inside the neuron.

It has long been suspected that Cannabis abuse increases risk of psychoses (Colizzi et al., 2016). In a mouse model of NMDAR antagonist-precipitated psychosis (MK-801), the CB1-selective antagonist AM 251 reduced behavioral symptoms, including hyperactivity (Kruk-Slomka et al., 2016). Interestingly, CB1-antagonism also improved psychosis-related memory impairments in this model, suggesting a possible role for endocannabinoid signaling in the memory deficits associated with schizophrenia and bipolar disorder.

#### AMPA receptors in CNS

An early Xenopus oocyte expression study examining sensitivity of various AMPA receptor subunit compositions demonstrated that high concentrations of anandamide (EC50 > 100 μM) effectively inhibit/s AMPA agonist-initiated currents (Akinshola et al., 1999). Anandamide inhibition was not reversed by SR, as expected in the CB1-deficient expression system, and similar efficacy was not observed for the synthetic agonist WIN. Enhancement of anandamide's effect was produced by addition of forskolin, a direct adenylyl cyclase activator, and partially reversed by cyclase inhibitors, implicating this enzyme in mediating the effect.

An interesting physiological interaction between endocannabinoid and AMPA receptor signaling has been reported in chicken embryo spinal cord (Gonzalez-Islas et al., 2012). In this system, a basal endocannabinoid tone inhibits activity of motor neurons which decreases spontaneous activity. Reversal of endocannabinoid tone via application of the CB1-selective antagonist, AM 251 resulted in an increase of spontaneous activity that, over 2 days, resulted in AMPA receptor desensitization, implicating endocannabinoid signaling in regulation of motor activity during development.

#### Metabotropic glutamate receptors in CNS

Physiological interaction of metabotropic glutamate receptors (mGluR) and endocannabinoid signaling has been reported in striatal and hippocampal slice preparations (Jung et al., 2005). In this system, activation of the mGluR5 receptor subtype resulted in release of the endocannabinoid 2-AG but not anandamide. This finding implicates 2-AG as the endocannabinoid involved in mediating AMPA receptor-induced plasticity important to processes of learning and memory.

### Inhibitory amino acid receptors in CNS

GABA and glycine are amino acid transmitters that activate inhibitory chloride channels, and in the case of GABA_B_, G_i/o_-coupled metabotropic receptors that activate inhibitory inwardly rectifying K^+^ flow. Overall sedative effects of cannabinoid agonists made positive regulation of these inhibitory amino acid transmitter systems seem likely, although clear evidence of interaction with GABAergic signaling systems has emerged only recently.

#### Gaba receptors in CNS

In the case of cannabinoid receptor-independent effects on GABAergic signaling, in hippocampal slice preparations it has been found that WIN55212-2 and anandamide (but not 2-AG) potentiate GABA release within rat dentate gyrus. This release promoted measurable inhibitory post-synaptic currents (IPSCs) measured by electrophysiology. The fact these WIN55212-2 and anandamide-promoted currents were resistant to both CB1- and CB2-selective antagonists in tissues taken from CB1 deficient mice, is evidence that they are the result of interaction with a yet uncharacterized target (Hofmann et al., 2011). Interestingly, the agonist specificity (WIN55212-2 and anandamide) of this IPSC effect is similar to that discovered earlier in GTPγS binding assays employing mouse tissue (Breivogel et al., 2001) suggesting that the same target is responsible.

In the context of behavior, 2-AG has been shown to reduce locomotor activity in CB1/CB2 double knockout mice (Sigel et al., 2011). Unfortunately only endocannabinoids were investigated in this study leaving open the question of WIN55212-2 activity that is implicated by earlier studies mentioned above. Hypermotility was observed in mice deficient for the GABA_A_ B_2_ subunit. When heterologously expressed in Xenopus oocytes, it was found that electrophysiological effects of 2-AG were only observed in systems producing B_2_ protein, suggesting this subunit is required for 2-AG interaction with GABA_A_. B_2_/2-AG interaction has been further supported by modeling and studies of the effect of amino acid substitutions (Baur et al., 2013). This work has recently been extended to a slice preparation model that demonstrates 2-AG influences GABA_A_ signaling under physiologically-relevant conditions (Golovko et al., 2014). This physiological modulation involves both synaptic and extrasynaptic populations of GABA_A_ receptors enhancing the former and inhibiting the latter in a manner to potentiate overall GABAergic inhibition (Brickley and Mody, 2012).

Endocannabinoid regulation of spinal nociceptive vs. non-nociceptive transmission has been established in vertebrate species (Pernía-Andrade et al., 2009). This work has been extended to demonstrate that the endocannabinoid 2-AG produces similar effects in a non-cannabinoid receptor-expressing invertebrate species of leech (Higgins et al., 2013). The target of 2-AG in this animal appears to be a TRPV channel, as a selective antagonist blocked the effect. Interestingly, the 2-AG effect to potentiate non-nociceptive vs. nociceptive transmission was antagonized by the GABA_A_-selective agent, bicuculline, suggesting a potential TRPV-GABAergic relationship in this physiological system.

#### Glycine receptors in CNS

Evidence for direct interaction of the endocannabinoids anandamide and 2-AG and glycine receptors were first reported from electrophysiological studies employing primary neuronal cell cultures and hippocampal slice preparations (Lozovaya et al., 2005). These experiments demonstrated that anandamide, 2-AG and the synthetic agonist WIN55212-2 inhibit glycine receptor conductance post-synaptically, and accelerate desensitization. This effect contrasts with the retrograde, presynaptic endocannabinoid mechanism that has been better characterized to date. Glycine receptor inhibition was observed in the presence of CB1, CB2, and vanilloid receptor antagonists, suggesting a direct agonist mechanism.

Complicating the picture, in a different *Xenopus* oocyte expression system, anandamide *agonism* of glycine receptors was discovered (Hejazi et al., 2006). These studies extended the effect to include the phytocannabinoid, THC. In an additional heterologous expression system, using HEK293 cell cultures expressing various glycine receptor subunits, an even more complex picture emerged (Yang et al., 2008). In these studies, anandamide and HU210 potently activated α1 subunit-containing glycine receptors. In contrast, the synthetic agonists HU210, HU308, and WIN55212-2 each potently (EC50 38—3030 nM) inhibited the α2 subunit-mediated currents, while the endocannabinoid NAGly (discussed above as a GPR18 agonist) activated it at high concentration. Efficacy and potency of glycine receptor inhibition varied with differential subunit expression. These types of apparent conflicts in pharmacological literature are common and, as we see with glycine receptors, are often attributable to different physiological/expression systems employed (Urban et al., 2007).

### Cholinergic receptors in CNS

Anecdotes have long suggested interaction between cannabinoid and nicotinic cholinergic signaling systems. This relationship is supported by behavioral (Pryor et al., 1978) and more recently biochemical evidence that demonstrates nicotine modulates multiple effects of the phytocannabinoid, THC (Valjent et al., 2002).

A direct mechanism underlying cannabinoid interaction with nicotinic receptors has been revealed through a series of *Xenopus* oocyte expression studies employing the endocannabinoid anandamide. The first of these found that anandamide potently inhibits nicotinic receptors comprised of α7 subunits (IC50 ~ 30 nM, Oz et al., 2003). This inhibition was not altered by CB1 or CB2 antagonists or cAMP modulating agents suggesting a direct nicotinic receptor interaction. Anandamide inhibition of α7-mediated currents didn't alter nicotine potency, only efficacy, indicating a non-competitive antagonist mechanism. The second study expanded the array of cannabinoid agonists employed in the system finding that only the endocannabinoids anandamide and 2-AG produce potent inhibition (Oz et al., 2004). Notably, THC, WIN, CP55940 did not. The final papers in this series found that the potency of inhibitory effects of both anandamide and ethanol are increased by their co-administration (Oz et al., 2005) and that anandamide potently and non-competitively also antagonizes receptors comprised of α4β2 subunits (EC50 ~ 50 nM, Spivak et al., 2007). Also, the phytocannabinoid CBD was found to inhibit nicotinic currents, albeit less potently (EC50 ~ 11 μM, Mahgoub et al., 2013).

More recently, these studies have been extended out of the oocyte system and into *in vivo* models. In the first of these, the non-hydrolysable analog of anandamide, methanandamide, was found to effectively antagonize nicotinic receptors present in cardiac post-ganglionic sympathetic neurons (Baranowska et al., 2008). In a second anesthetized rat model, nicotine infusion increased activity of dopaminergic neurons in the ventral tegmental area, a region important to reward-related release of dopamine in nucleus accumbens. Nicotine stimulation of this activity was found to be reversed by URB597, an indirect cannabinoid agonist that inhibits anandamide metabolism (Melis et al., 2008). This effect did not extend to the hydrolysis-resistant methanandamide, suggesting that a product of an alternate anandamide metabolic pathway is responsible. Interestingly, both oleoylethanolamine (OEA) and palmitoylethanolamide (PEA) were found to have efficacies similar to anandamide. As OEA and PEA are known agonists of peroxisome proliferator-activated receptor-α (PPARα) activation of this receptor was studied as the potential mechanism for nicotinic receptor inhibition. Results revealed that PPARα activation promotes kinase activity that increases nicotinic receptor phosphorylation and inactivation. Whether the efficacy of anandamide, perhaps through increasing availability of ethanolamine precursors, depends upon a similar PPARα-related mechanism remains an open question.

### Nuclear receptors in cancer

#### Peroxisome proliferator-activated receptors (PPAR) in cancer

PPARs are a superfamily of nuclear receptors that play an important role in the regulation of lipid metabolism, glucose homeostasis, cell differentiation and tumorigenesis (Vamecq and Latruffe, 1999). PPAR transcription factors form heterodimers with retinoid X receptor (RXR) that then bind to peroxisome proliferator responsive elements (PPRE) and regulate transcription of target genes. PPARs are classified into three subtypes: PPARα, PPARδ, and PPAR⋎. In cancer cells, targeting PPAR⋎ with selective agonists inhibits cell proliferation, induces programmed cell death (apoptosis and autophagy) and promotes cell differentiation in multiple *in vitro* and *in vivo* studies (Campbell et al., 2008).

#### PPARγ in cancer

Cannabinoids increase PPAR⋎ transcriptional activity by diverse mechanisms (reviewed in O'Sullivan, 2007). Cannabinoids can bind and activate cell surface cannabinoid receptors which promotes MAPK signal transduction resulting in downstream PPARγ activation. Cannabinoid-induced PPARγ activation can also occur independent of cannabinoid receptors by direct and indirect mechanisms. Cannabinoids (e.g., ajulemic acid) and endocannabinoids (e.g., AEA and 2-AG) can directly bind, and increase the transcriptional activity of, PPARγ. Indirect PPARγ activation occurs as a consequence of the metabolism of endocannabinoids (2-AG, AEA) to PPARγ agonists or by increasing the synthesis of endogenous PPAR agonists (Burstein, 2005; O'Sullivan, 2007).

Several studies have shown that PPAR⋎ mediates the antitumor activity of cannabinoids independent of CB1 and CB2 receptors. In lung cancer cells, PPARγ activation was required for CBD-induced apoptosis (Ramer et al., 2013). This effect of CBD on PPARγ was mediated by production of the prostaglandins (PGs), PGD_2_ and 15deoxy, Δ^12, 14^ PGJ2 (15d-PGJ_2_) which are PPARγ activators. Prevention of PG synthesis with a selective inhibitor of COX-2 activity (NS-398) or siRNA directed toward COX-2 reduced CBD-induced PPARγ nuclear translocation and cell death. However, the use of CB1 (AM-251), CB2 (AM-630), or TPRV1 (capsazepine) antagonists did not alter CBD-mediated apoptotic cell death. Furthermore, in human cervical carcinoma cells treated with the non-hydrolysable AEA derivative, met-AEA, apoptosis was reliant upon the production of PGD_2_ and PGJ_2_ as well as the activation of PPARγ (Eichele et al., 2009). These findings indicate that cannabinoid-induced prostaglandin synthesis and the engagement of these prostaglandins with PPARγ represents an important pathway by which cannabinoids regulate cellular fate independent of the cannabinoid receptors.

### Ion channels in CNS and cancer

Direct cannabinoid modulation of the activity of ionotropic receptors have been discussed according to ligand type in the section above. In terms of interaction with non-ligand gated ion channels, cannabinoid receptor-stimulated G-proteins have long been known to inhibit voltage-gated calcium channels and to activate inward-rectifying potassium channels, consistent with inhibition of transmitter release (Shen et al., 1996). In addition to ligand-gated channel effects, evidence for direct cannabinoid interaction with other non-ligand-gated ion channels has emerged and is discussed below.

#### Voltage-gated sodium channels in CNS

In the case of the synthetic cannabinoid, ajulemic acid that is structurally-related to THC, patch clamp studies of HEK293 and ND7/23 cells expressing various types of voltage-gated sodium channels demonstrated inhibition with potencies ranging from 1 to 10 μM (Foadi et al., 2014). These results suggest that the efficacy of ajulemic acid to reduce neuropathic pain may involve sodium channel inhibition.

#### Transient receptor potential (TRP) cation channels in CNS

TRP channels are a superfamily of cation channels located on the cell plasma membrane that control inward movement of monovalent or divalent cations notably including Ca^++^. These channels are largely sensory-related, and they are particularly important to perception of temperature and mechanical stimulation (Voets et al., 2005). TRP channels have already been mentioned above (in the context of anandamide interaction with NMDA receptors) (section NMDA Receptors in CNS). In addition to a role in mediating peripheral afferent signaling, TRP channels are physiologically relevant to the extraneuronal function of vascular smooth muscle (Fernandes et al., 2012) and are also expressed within CNS (Vennekens et al., 2012). Several members of this family may represent “ionotropic cannabinoid receptors” moving cannabinoid signaling into line with most other CNS signaling systems that include both metabotropic and ion channel targets (Akopian et al., 2009).

Several members of this family, including TRP ankyrin type 1 (TRPA1), the vanilloid receptors types 1 and 2 (TRPV1 and TRPV2), and TRP melastatin type 8 (TRPM8), have been identified through a remarkably ambitious and thorough screening study as targets of an array of compounds isolated from *Cannabis* (De Petrocellis et al., 2011). These phytocannabinoids include cannabichromene (CBC), CBD, cannabidiol acid (CBDA), cannabidivarin (CBDV), cannabidivarin acid (CBDVA), cannabigerol (CBG), cannabigerol acid (CBGA), cannabigivarin (CBGV), cannabinol (CBN), THC, THC acid (THCA), and tetrahydrocannabivarin acid (THCVA). Each of these compounds are effective agonists for TRP1A with EC50s ranging from 90 nM to 8.4 μM and a rank order of potency of CBC > CBD > CBN > CBDV > CBG > THCV > CBGV > THCA > CBDA > CBGA > THCVA. At TRPV1; CBD, CBG, CBN, CBDV, CBGV, and THCV have agonist activity with EC50 < 10 μM (the most potent compound being CBD with EC50 = 1 μM). At TRPV2, several of the compounds had agonist activity with a rank order of potency = THC > CBD > CBGV > CBG > THCV > CBDV > CBN (EC50 ranging from 650 nM to 19 μM). Finally, at TRPM8, each of the compounds were found to effectively *antagonize* icilin-stimulated Ca^++^ conductance with a rank order of potency = CBD > CBG > THCA > CBN > THCV > CBDV > CBGA > THCVA > CBGV > CBDA and IC50s ranging from 60 nM to 4.8 μM.

Interestingly, the synthetic cannabinoid compounds AM251 and AM630, that are CB1- and CB2-selective antagonists/inverse agonists, respectively, have been found to activate Ca^++^ channels in primary cultures of trigeminal nerve neurons (Patil et al., 2011). When evaluated in CHO cell culture systems heterologously expressing TRP1A and TRPV1 channels, it was found that both CB receptor antagonists have agonist activity at TRP1A but not TRPV1. These findings suggest that *in vivo* AM251 and AM630 may activate sensory neurons via TRP1A agonism.

##### Focus on TRPV1 in CNS

Also known as capsaicin receptors or vanilloid receptors, these promiscuous cation channels are both temperature- and pH-sensitive. They are also capable of ligand activation, notably by capsaicin (the active constituent of spicy peppers). Shortly after its isolation and identification as the first endogenous cannabinoid present in brain, anandamide was found to interact with a non-cannabinoid receptor target: TRPV1 (Zygmunt et al., 1999). The relationship between anandamide and other endocannabinoid signaling and TRPV1 channel activity has been the subject of excellent recent reviews (Dainese et al., 2010; Marzo and Petrocellis, 2010; Di Marzo and De Petrocellis, 2012), and so, only two notable recent additions, confirming the physiological relevance of TRPV1 agonism by anandamide to neuronal activity, will be presented here.

The first is an interesting recent study of the analgesic effects of the fatty acid amide hydrolase (FAAH) inhibitor: URB597. This compound reduces metabolism of the endocannabinoid, anandamide, thereby enhancing effects of endogenous release. Systemic administration of URB597 effectively reduces measures of neuropathic pain in the rat chronic constriction injury model (Starowicz et al., 2013). Analgesic efficacy of URB597 was maintained in the presence of the CB1-selective antagonist, AM251. However, pretreatment of animals with the potent TRPV1 antagonist, iodoresiniferatoxin, reversed effects of URB597. These important results reinforce the role of endocannabinoid signaling in pain sensation, and they demonstrate that agonism of the TRPV1 subtype of vanilloid receptors is important to this type of analgesic efficacy of anandamide.

A second recent study employed mouse hippocampal cultures of CA1 presynaptic CB1-expressing GABAergic neurons and post-synaptic pyramidal neurons. Activities of both neuron types were studied simultaneously via an ambitious paired patch clamping technique (Lee et al., 2015). As expected, post-synaptic depolarizations were associated with increased 2-AG release and diffusion, ultimately resulting in presynaptic CB1 activation. In the case of perisomatic input to pyramidal cells, a basal tone of 2-AG stimulation of presynaptic CB1 receptors was found to maintain a “set point” of GABA release. This set point was revealed by increased inhibition following application of JZL184, an inhibitor of MAGL. As MAGL is the principal enzyme responsible for degrading 2-AG, inhibitors like JZL184 act as indirect agonists. Interestingly, addition of PF3845, an inhibitor of the enzyme FAAH responsible for degrading anandamide, effectively reduced 2-AG tone, suggesting an antagonistic relationship between the two endocannabinoids in regulating GABAergic signaling in hippocampus. The indirect anandamide agonist effect of PF3845 was reversed in the presence of AMG9810, a selective TRPV1 inhibitor, demonstrating that anandamide effectively modulates basal 2-AG tone via activation of this post-synaptic cation channel, presumably via reduced activity of the enzyme responsible for 2-AG synthesis, DAGLα.

#### TRP cation channels in cancer

A growing body of evidence implicates members of the TRP family, including TRPV1, TRPV2, and TRPM8, in calcium-mediated signal transduction that regulates proliferation, migration, and metastasis of cancer cells (reviewed by Déliot and Constantin, 2015; Yee, 2015). As discussed previously, numerous cannabinoids bind to, and modify, the activity of TRP channels. We review TRP-mediated activity of several cannabinoids in cancer below.

##### Vanilloid receptor type 1 (TRPV1) in cancer

TRPV1 Ca^++^ channels are located on the plasma membrane and other subcellular organelles including the endoplasmic reticulum (ER). TRPV1 regulates intracellular Ca^++^ levels by controlling Ca^++^ movement across the plasma membrane and its release from the ER and sarcoplasmic reticulum (Gallego-Sandín et al., 2009). Establishing the relationship between TRPV1 expression levels, and characteristics such as the tumor grade (predicted aggressiveness of the tumor) or tumor stage (tumor size and degree of spread), aids in determining its potential role in cancer. It has been reported that TRPV1 expression increased with increasing tumor grade in prostate cancer biopsies (Czifra et al., 2009), but it was inversely correlated with tumor stage in bladder cancer (Lazzeri et al., 2005) suggesting that the relationship between TRPV1 expression and tumor behavior may be tumor-type specific. Other studies provide evidence that TRPV1 activation is necessary for cannabinoid-induced tumor death. The endocannabinoid, AEA, decreased the viability of cervical cancer cells that overexpressed TRPV1, CB1, and CB2. In these cells, the antiproliferative effect of AEA was counteracted by blockade of TRPV1 but not by antagonism of the CB1 or CB2 receptors (Contassot et al., 2004a). Similarly, AEA initiated TRPV1-dependent, CB receptor-independent apoptosis in human glioma cells (Contassot et al., 2004b). Based upon these interesting findings, further investigation should uncover roles of TRPV1 in cancer and identify other cannabinoid agonists that decrease cancer growth by targeting this pathway.

##### Vanilloid receptor type 2 (TRPV2) in cancer

The specific role of TRPV2 in carcinogenesis appears to differ according to tumor type. In urothelial carcinoma, TRPV2 expression increased with increasing tumor stage and grade (Caprodossi et al., 2008). However, in hepatocellular carcinoma, TRPV2 expression was lower in poorly differentiated (compared to well-differentiated) tumors (Liu et al., 2010). In glioblastoma multiforme, elevated TRPV2 expression correlated with increased patient survival (Alptekin et al., 2015) while increased TRPV2 expression was associated with poor survival in esophageal squamous cell carcinoma (Zhou et al., 2014). Although the aforementioned studies demonstrate that the role of TRPV2 in cancer progression is unclear, research that examined the impact of cannabinoids on this cation channel demonstrated that TRPV2 activation decreased tumor cell survival and sensitized tumor cells to clinically available chemotherapeutic agents. The phytocannabinoid, CBD, increases inward movement of Ca^++^, but it has low affinity interactions with CB1 and CB2 receptors (De Petrocellis et al., 2011; McPartland et al., 2015). In glioblastoma, CBD increased the plasma membrane expression of TRPV2 and prevented cell resistance to carmustine (BCNU), doxorubicin, and temozolmide (Nabissi et al., 2013). This sensitization to cytotoxic chemotherapeutics was prevented by siRNA-mediated disruption of TRPV2 expression. Consistent with this finding, the antiproliferative effects of CBD in stem-like glioma cells were reversed in the presence of the Ca^++^ channel blocker, ruthenium red (RR), and the selective TRPV2 blocker, tranilast (Nabissi et al., 2015). As anticipated, antagonists of CB1 (AM251) and CB2 (AM630) were not able to rescue cells from cell death (Nabissi et al., 2015). Moreover, the sensitivity of multiple myeloma cells to bortezomib was heightened by cell exposure to CBD in a TRPV2-dependent manner (Hashimoto et al., 1986). These findings suggest calcium mobilization regulated by TRPV2 is essential for CBD-mediated cell death. Further investigation of the activity of TRPV2 in cancer is needed to determine the feasibility of utilizing cannabinoids to target this cation channel as a therapeutic strategy.

### Transporters in CNS

Transporters function to actively facilitate movement of molecules across membranes against concentration and osmotic gradients. Drugs that target transporters exert effects through altering distribution of other molecules that, in turn, alter membrane potential of excitable membranes and/or terminate action of transmitters that are regulated by uptake. With respect to cannabinoid signaling, evidence suggests that transport may contribute to anandamide signal termination, in addition to metabolism by FAAH (Nicolussi and Gertsch, 2015). The anandamide transporter remains a target for development of selective inhibitors (Nicolussi et al., 2014).

Adding to the promiscuous interaction of anandamide with non-CB1/CB2 targets is evidence for inhibition of a Na^+^/Ca^++^ exchange pump (Kury et al., 2014). This inhibition was of both influx and efflux, and was not altered by CB1- and CB2-selective antagonists, pertussis toxin or guanyl nucleotide analogs, consistent with a direct interaction with the transporter. Although this transporter is most relevant to cardiac function, similar transporters are important to CNS activity, and these results suggest potential anandamide neuronal efficacy related to ion transport.

Part of the cannabinoid effects on glycinergic signaling discussed above (section Glycine Receptors in CNS) may in part be attributable to interaction with glycine transporters. In the case of glycine transporter GLYT1a, arachidonic acid and anandamide have interesting opposing modulatory actions (Pearlman et al., 2003). Interestingly, evidence that upregulation of GLT-1, the transporter for the excitatory transmitter glutamate, in preventing cannabinoid dependence, is also emerging (Gunduz et al., 2011).

As discussed above in the context of adenosine (section Adenosine Receptors in CNS), evidence demonstrates that the phytocannabinoid CBD antagonizes an adenosine transporter, reducing inflammatory responses (Carrier et al., 2006). This activity was absent in adenosine A_2A_ knockout mice, and reversed by a selective antagonist, implicating indirect A_2A_ agonism, via inhibition of transporter-mediated signal termination, as the mechanism of CBD-mediated immunosuppression.

While not demonstrating a direct interaction, studies of effects of the synthetic CB1/CB2 agonist, WIN, have revealed that dosages effective in reducing locomotor activity (0.1–1 mg/kg) also decrease expression of dopamine transporters (Fanarioti et al., 2015). Transporter densities were measured by *in situ* [^3^H]-WIN35428 binding. Decreased levels were observed in several brain regions relevant to drug abuse including: nucleus accumbens core and shell; substantia nigra and; ventral tegmentum.

### Enzymes in CNS and cancer

#### Endocannabinoid synthesis and metabolism in CNS

The elaborate study of phytocannabinoids discussed above in the context of TRP channels [section Transient Receptor Potential (TRP) Cation Channels in CNS] in CNS also included evaluation of the same series of compounds for effects on activity of enzymes related to endocannabinoid signaling—including those responsible for endocannabinoid production (DAGLα), metabolism [monoacylglycerol lipase (MAGL), fatty acid amide hydrolase (FAAH), and N-acylethanolamine acid amide hydrolase (NAAA)] and uptake (anandamide cellular uptake [ACU]). Results demonstrated that a few of the compounds inhibit DAGLα with a rank order of potency CBDV = CBDA > CBGA = THCA = CBDVA (EC50s ranged from 17 to 35 μM, De Petrocellis et al., 2011). Both CBC and CBG partially inhibit MAGL activity with efficacies of ~50% and IC50s = 50 and 95 μM, respectively. THCA is a more fully-effective MAGL inhibitor with IC50 = 46 μM. Only CBD was found to inhibit FAAH at concentrations under 50 μM (IC50 = 15 μM). Only CBDA inhibited NAAA, the enzyme responsible for degradation of the endocannabinoid-like compound N-palmitoylethanolamine (PEA), doing so with IC50 = 23 μM.

#### Fatty acid amide hydrolase (FAAH) in cancer

FAAH is a membrane-bound serine hydrolase that catabolizes naturally occurring fatty acid amides including AEA, OEA, and PEA to fatty acids plus ethanolamine (Cravatt et al., 1996, 2001). FAAH regulates the levels of its main cannabinoid substrate AEA, and it is overexpressed in different types of cancer. FAAH expression was elevated in lung adenocarcinoma compared to non-malignant respiratory epithelial cells (Ravi et al., 2014). In prostate cancer cells, FAAH was overexpressed, and the upregulated FAAH levels correlated with poor patient prognosis (Thors et al., 2010). In addition, AEA levels and FAAH expression and activity were elevated in human colorectal cancer tissue compared to non-tumor colon tissue (Chen et al., 2015). Consistent with findings demonstrating that FAAH is overexpressed in cancer, *in vitro* and *in vivo* studies revealed that the antitumor activity of endocannabinoids was increased by inhibiting FAAH activity. AEA-mediated death in HT29 colorectal cancer cells was enhanced by its co-administration with the FAAH inhibitor, MAFP (Patsos et al., 2005). Similarly, the apoptotic effect of AEA was increased by co-exposure to the FAAH inhibitor URB597 in non-melanoma skin cancer cells (Kuc et al., 2012). In addition, FAAH inhibition with URB597 prevented AEA degradation and increased its cytotoxicity in neuroblastoma cells (Hamtiaux et al., 2011). The reduction in cell viability caused by the co-administration of AEA and URB597 was found to be independent of CB1, TRPV1, PPAR-α, PPAR-γ, and GPR55 receptor activity (Hamtiaux et al., 2011). These findings suggest that increasing cellular levels of AEA, by decreasing FAAH activity, allows greater quantities of this endocannabinoid to interact with its molecular targets (thereby enhancing its antitumor activity).

#### Monoacylglycerol lipase (MAGL) in cancer

MAGL is a membrane-associated serine hydrolase that metabolizes monoacylglycerols (MAG) to free fatty acids (FFA) plus glycerol. MAGL is a prominent regulator of the levels of the endocannabinoid, 2-AG. MAGL was found to be elevated in aggressive cancer cells (compared to non-aggressive cells) (Nomura et al., 2010). MAGL increased FFA production to promote tumor growth, tumor cell migration, and tumor invasion. Inhibiting MAGL expression with shRNAs, or blocking its activity with JZL184, reduced tumor cell migration in a manner that was not dependent on the CB1 or CB2 receptors (Nomura et al., 2010). These data indicate that the tumor promoting metabolic products of 2-AG increase cancer cell survival. As such, examining MAGL, 2-AG and FFA levels in different tumor types, and identifying cellular targets of FFAs, will be an important step toward understanding whether this pathway can be exploited for therapeutic benefit.

#### Cycloxygenase-2 (COX-2) in cancer

COX-2 oxygenates arachidonic acid (AA) to prostaglandin H_2_ (PGH_2_) that is further metabolized by PG synthase-E, -F_2α_, and -D to PGE_2_, PGF_2α_, and PGD_2_, respectively (Rouzer and Marnett, 2009). PGD_2_ is then dehydrated to J-series PGs. Substantial evidence points to a role for COX-2 and its products, notably PGE_2_, in the growth of epithelial tumors of the colon, lung, breast, skin, and other organs (Jiao et al., 2014; Ma et al., 2015; Majumder et al., 2015; Kiraly et al., 2016). Pro-inflammatory COX-2 and PGE_2_ promote cell proliferation, angiogenesis, and cell migration by activating PGE_2_ receptors which increase oncogene and cytokine activity (Rundhaug et al., 2007, 2011). In contrast, cannabinoids possess anti-inflammatory activity (Burstein and Zurier, 2009). The mechanism by which cannabinoids suppress inflammatory cascades is a matter of debate; although, some studies suggest that cannabinoids block cyclooxygenase activity. Ruhaak et al. reported that several cannabinoids isolated from *Cannabis sativa* inhibited COX-2 activity and reduced prostaglandin generation (Ruhaak et al., 2011). Tetrahydrocannabinolic acid (THC-A), cannabigerol (CBG), and cannabigerolic acid (CBGA) inhibited the activity of COX-2 in a cell-free enzyme assay with IC50 values of 6.3 × 10^−4^, 2.7 × 10^−4^, and 2.0 × 10^−4^ M, respectively. However, in TNFα stimulated cancer cells, a significant reduction in COX-2 activity was not observed (Ruhaak et al., 2011). In a different study, CBDA was a potent and selective inhibitor of COX-2 activity with an IC50 value of 2.2 μM and an IC50 value of 20 μM for COX-1 (Takeda et al., 2008). These authors suggested that the inhibitory effect of cannabinoids on COX-2 activity was due to the presence of the salicylic acid moiety in the chemical structure of CBDA. Additional studies are needed to uncover the biological and clinical relevance of cannabinoid-mediated cyclooxygenase inhibition.

In contrast to the inhibitory effects of cannabinoids on COX-2 activity mentioned previously, other reports indicate that cannabinoids upregulate COX-2 expression. A series of studies by Hinz et al. demonstrated that cannabinoid-induced COX-2 expression increased the synthesis of arachidonic acid-derived prostaglandins that promoted cell death (Hinz et al., 2004a,b). In lung cancer cells, CBD increased COX-2 expression and the synthesis of D- and J- series prostaglandins which initiated tumor cell apoptosis (Ramer et al., 2013). Met-AEA also increased COX-2 expression in a manner that was dependent on lipid rafts, ceramide, and the activation of p38 and p42/44 MAPK (Ramer et al., 2003; Hinz et al., 2004a). However, Met-AEA-induced apoptosis was not reversed by CB1 (AM251), CB2 (AM630), or TRPV1 (capsazepine) receptor antagonists in human neuroglioma cells (Hinz et al., 2004b). It was also reported that Met-AEA increased ceramide synthesis, COX-2 expression and the production of proapoptotic PGD_2_ in human cervical carcinoma cells (Eichele et al., 2009). In this study, inhibiting the production of D-series PGs using siRNA directed against lipocalin-PGDS or siRNA against PPARγ prevented Met-AEA-induced apoptosis. A reversal in Met-AEA-induced apoptosis, however, was not observed in the presence of the receptor antagonists, AM251, AM630 or capsazepine (Eichele et al., 2009). In contrast, Garder et al. determined that Met-AEA increased lung tumor growth *in vitro* and *in vivo* (Gardner et al., 2003). This pro-tumorigenic effect was blocked by pharmacological inhibitors of COX-2, p38, and p42/44, but it was not affected by the CB1 (SR141716) and CB2 (SR144528) receptor antagonists (Gardner et al., 2003).

AEA is also a substrate of COX-2, and, as a result, is cytotoxic in tumor cells that overexpress COX-2. AEA has an unmodified arachidonate backbone, and it is therefore susceptible to oxidative metabolism by COX-2 to form prostaglandin-ethanolamines, also known as prostamides (PM) (Yu et al., 1997; Kozak et al., 2002; Soliman et al., 2016). The metabolic products of AEA, prostamides-E_2_, F_2α_, and -D_2_, do not bind to prostaglandin receptors, and they are metabolically stable relative to prostaglandins derived from arachidonic acid (Kozak et al., 2001; Matias et al., 2004). Recently, we demonstrated that AEA was also metabolized by COX-2 to novel J series prostamides that initiated tumor cell apoptosis (Kuc et al., 2012; Ladin et al., 2017). Because epithelial cancer cells typically overexpress COX-2, AEA was metabolized to pro-apoptotic J-series prostamides in tumor cells, but J-series prostamides were not detected in non-tumor cells which had low endogenous levels of COX-2 (Soliman et al., 2016). Blockade of AEA degradation with the FAAH inhibitor, URB597, increased J-series prostaglandin synthesis and apoptosis; however, inhibition of CB1, CB2, and TRPV-1 receptors using selective antagonists did not reverse this effect (Soliman and Van Dross, 2016). Furthermore, cell treatment with exogenous 15-deoxy, Δ^12, 14^ prostamide J_2_ (15d-PMJ_2_), the most abundant J-series product of AEA metabolism by COX-2, also caused cell death *in vitro* and *in vivo* (Ladin et al., 2017). Reports by Pastos et al. also demonstrated that COX-2 was required to induce death in cancer cells treated with AEA (Patsos et al., 2005, 2010). Prostamides E_2_ and D_2_ were noted to cause tumor cell death while blockade of COX-2 activity or expression partially reversed this antiproliferative effect. However, AEA-mediated cell death was not blocked by CB1, CB2, or TRPV1 receptor antagonists (Patsos et al., 2010). Thus, COX-2 is a critical regulator of CB receptor-independent cannabinoid activity in cancer cells. These studies demonstrate that cannabinoid metabolism and signal transduction is modulated by COX-2 and that COX-2 is also inhibited by molecules within the cannabinoid family. This implies that a complex relationship exists between cannabinoids, the endocannabinoid system, and cyclooxygenases in cancer and potentially in other organ systems.

#### Phosphatases in cancer

The restoration of phosphatase expression and activity is a desirable effect of chemotherapeutic agents, as these enzymes dephosphorylate kinases, and other pro-tumorigenic proteins, that are often constitutively active in cancer (Perrotti and Neviani, 2013). The anticancer activity of WIN55212-2 was reported to be mediated by phosphatases in a cannabinoid receptor-independent manner (Sreevalsan et al., 2011; Sreevalsan and Safe, 2013). WIN55212-2 increased the expression of several phosphatases including dual specificity phosphatases 1 and 10 (DUSP1, DUSP10) and protein tyrosine phosphatase, non-receptor type 6 (PTPN6) in LNCaP prostate cancer cells (Sreevalsan et al., 2011). WIN55212-2 also increased PTPN6 expression and protein phosphatase A2 (PPA2) activity in SW480 colon cancer cells (Sreevalsan et al., 2011; Sreevalsan and Safe, 2013) and reduced the expression of the pro-tumorigenic transcription factors, specificity protein 1, 3, and 4 (SP1, SP3, and SP4). The effect of WIN55212-2 on SP expression and apoptosis was dependent on phosphatase PPA2, a prominent serine-threonine phosphatase in eukaryotic cells that regulates the cell cycle and apoptosis. Specifically, it was determined that WIN55212-2 increased the activity of PPA2 and activation of the Sp repressor, ZBTB10, thereby inhibiting Sp expression through a pathway that was regulated by micro RNA-27a (miR-27a). However, this cytotoxic activity was not prevented by blockade of CB1 or CB2 receptor activity (Sreevalsan and Safe, 2013). These reports suggest that cannabinoid activity is modulated by phosphatases independent of the cannabinoid receptors. Phosphatases have significant impacts on cellular behavior, because these enzymes control kinase activity. Therefore, examination of the role of phosphatases in cannabinoid activity may allow these proteins to be targeted to improve the action of cannabinoids.

### Other non-receptor targets in cancer

#### Lipid rafts and ceramide

Lipid rafts are glycoprotein microdomains enriched in cholesterol and sphingolipids. Lipid rafts serve as organization centers that promote interactions between proteins to aid in intracellular signal transduction (Simons and Ikonen, 1997). Ceramide is a membrane lipid that displaces cholesterol from lipid rafts, enhances membrane rigidity, stabilizes rafts and induces formation of large raft domains (“platforms”) in plasma membranes (Simons and Ikonen, 1997; Megha and London, 2004). It has been reported that the antitumor activity of cannabinoids is regulated by ceramide and lipid rafts which control CB1 and/or CB2-mediated signal transduction (Bari et al., 2005; Sarnataro et al., 2006). However, other studies demonstrate that ceramide and lipid rafts transmit lethal cannabinoid signals independent of CB1 and CB2. DeMorrow et al., found that AEA increased ceramide synthesis and localization of the death receptor/ligand, Fas/FasL, to lipid rafts leading to cholangiocarcinoma cell death (DeMorrow et al., 2007; Huang et al., 2011). In these studies, selective antagonists of CB1 and CB2 did not inhibit the cytotoxicity of AEA. In a different report, lipid raft disruptors completely attenuated AEA-mediated cell death in neuroblastoma cells (Hamtiaux et al., 2011). This cytotoxicity was not blocked by pharmacological antagonism of CB1, CB2, GPR55, TRPV1, or PPAR⋎. Similarly, the disruption of lipid rafts, but not the antagonism of CB1, CB2, or TRPV1 receptors, prevented AEA cytotoxicity in cutaneous melanoma cells (Adinolfi et al., 2013). In addition, Sarker and Maruyama found that disruption of lipid rafts blocked AEA-induced oxidative stress and apoptosis independent of the CB1, CB2, or TRPV1 receptors in different cell lines (Sarker and Maruyama, 2003). Consistent with these findings, Met-AEA-induced apoptosis was inhibited by the use of ceramide synthase inhibitors and lipid raft disruptors but not by CB1, CB2, or TRPV1 receptor antagonists in human neuroglioma and cervical carcinoma cells (Hinz et al., 2004b; Eichele et al., 2009). Moreover, WIN55212-2, caused lipid raft-mediated cell death in cultured melanoma cells in a CB1/CB2 receptor-independent manner (Scuderi et al., 2011). Collectively, these findings suggest that cannabinoids reduce tumor cell viability by modulating lipid rafts through cannabinoid receptor-dependent and -independent pathways.

#### Oxidative stress in cancer

Reactive oxygen species (ROS) are second messenger signal transduction molecules in eukaryotic cells. Low levels of ROS are needed for physiological processes such as immune defense against pathogens, mitogenic responses, and maintenance of cellular homeostasis (Finkel, 1998; Kim et al., 1998; Dröge, 2002). Physiologic levels of ROS are maintained by a balance between antioxidant and pro-oxidant systems in the cell. However, when antioxidant systems become overwhelmed, oxidative stress occurs. Cancer cells contain supraphysiological levels of ROS that activate signaling pathways which promote cell proliferation, survival, angiogenesis, and metastasis (Nourazarian et al., 2014). However, excessively high ROS levels causes oxidative damage to cellular proteins, lipids, and DNA that triggers cell cycle arrest and cell death (Kaur et al., 2014). Structurally distinct cannabinoids can inhibit tumor cell survival by generating high levels of ROS. CBD decreased glutathione (GSH) levels and increased GSH reductase and GSH peroxidase content thereby causing cytotoxic oxidative stress in glioma cells (Massi et al., 2006). The cytotoxicity of CBD was abrogated by the antioxidant, α-tocopherol; however, blockade of CB receptor activity or ceramide synthesis did not significantly alter CBD cytotoxicity (Massi et al., 2004). Moreover, CBD-induced oxidative stress and cell death occurred in tumorigenic U87 glioma cells but not in non-tumorigenic primary glial cells (Massi et al., 2004), suggesting ROS elicits tumor-selective cytotoxicity. Ligresti et al. also found that antioxidants (α-tocopherol and vitamin C), but not CB receptor antagonists, blocked the antiproliferative activity of CBD in breast adenocarcinoma cells (Ligresti et al., 2006). Similarly, Shrivastava et al. determined that CBD increased ROS production that initiated autophagy and apoptosis independent of CB1, CB2, or TRPV1 in breast cancer cells (Shrivastava et al., 2011).

In several reports, the endocannabinoid, AEA, induced ROS-dependent, CB receptor-independent cell death. Oxidative stress and apoptosis in AEA treated non-melanoma skin cancer cells were prevented by the antioxidant, N-acetyl cysteine (NAC) (Van Dross, 2009). Blockade of CB1 and CB2 receptor activity did not rescue cells from AEA-mediated oxidative stress or apoptosis (Soliman and Van Dross, 2016). Similarly, in colon cancer cells, AEA-induced cell death and apoptosis was reversed by the use of antioxidants but not by CB1 or CB2 receptor antagonists (Gustafsson et al., 2009; Soliman, 2015). These findings suggests that oxidative stress regulates cannabinoid activity through pathways that circumvent the cannabinoid receptors.

#### Endoplasmic reticulum (ER) stress in cancer

ER stress occurs when the capacity of the cell to fold proteins is exceeded by the protein folding load resulting in the accumulation of unfolded proteins in ER (the unfolded protein response, UPR) (Holcik and Sonenberg, 2005). The UPR is an integrated intracellular signal transduction pathway that is regulated by three ER-resident stress sensors: double-stranded RNA-activated protein kinase (PKR)-like endoplasmic reticulum kinase (PERK), activating transcription factor-6 (ATF6), and inositol requiring kinase-1 (IRE1) (Lin et al., 2008; Chakrabarti et al., 2011). Activated PERK phosphorylates the α subunit of eukaryotic initiation factor 2α (eIF2α), resulting in a reduction in global translation with a preferential increase in the expression of activated transcription factor 4 (ATF4)-regulated genes (reviewed in Marciniak et al., 2006). Activation of PERK, ATF6, and IRE1 promotes cell survival by decreasing protein synthesis and by increasing protein folding and degradation to reestablish ER homeostasis. However, excessive or prolonged ER stress activates cell death pathways primarily by increasing expression of the transcription factor, C/EBP homologous protein 10 (CHOP10, also known as the growth arrest and DNA damage-inducible gene 153 [GADD153]) which transcribes pro-apoptotic genes (reviewed in Marciniak and Ron, 2006). Several studies have shown that the ER stress pathway mediates the anticancer activity of cannabinoids. The phytocannabinoid, THC, increased phosphorylation of eIF2α and the expression of the CHOP10 transcriptional product, TRB3, in hepatocellular carcinoma cells (HEPG2), human glioma cells, and RasV12/E1A-transformed mouse embryonic fibroblasts (MEF, Salazar et al., 2009, 2013; Vara et al., 2013). TRB3 is known to inhibit cancer cell proliferation by inactivating AKT/ mammalian target of the rapamycin complex 1 (mTORC) signal transduction (Ohoka et al., 2005). In human glioma cells treated with THC, it was determined that TRB3 was required for inhibition of Akt/mTORC1 signaling and the induction of autophagy and apoptosis (Salazar et al., 2009). Furthermore, unlike Trib3^+/+^ (also known as TRB3) MEF cells, Trib3^−/−^ MEFs were resistant to cell death caused by THC (Salazar et al., 2013). Similar results were observed when the two cell types were injected subcutaneously in nude mice that were subsequently treated with THC. THC reduced the growth of Trib3^+/+^ xenograft tumors; however, this effect was not seen in mice engrafted with Trib3^−/−^ cells (Salazar et al., 2013). WIN55212-2 also induced ER stress and increased the expression of CHOP10 and TRB3 in cancer cells (Wasik et al., 2011; Notaro et al., 2014; Pellerito et al., 2014). In addition, WIN55212-2 reduced the viability of osteosarcoma cells by initiating cytotoxic autophagy (Notaro et al., 2014). Genetic ablation of CHOP10 using siRNA prevented WIN-induced autophagic cell death. WIN55212-2 also caused autophagy and apoptosis in human colorectal cancer cells that was prevented by reducing the expression of CHOP10 with siRNA (Pellerito et al., 2014). Interestingly, Wasik et al. reported that WIN55212-2-induced autophagic cell death in Mantle cell lymphoma (MCL) cells occurred independent of the CB1 and CB2 receptors (Wasik et al., 2011). Consistent with these observations, the ER stress inhibitors, phenylbutyric acid (PBA) and salubrinal, prevented apoptosis in non-melanoma skin cancer cells treated with AEA. However, selective antagonism of the CB1 and CB2 receptor failed to inhibit AEA-induced ER stress or apoptosis (Soliman and Van Dross, 2016; Soliman et al., 2016). Structurally diverse cannabinoids initiate cytotoxic ER stress in cancer cells independent of the cannabinoid receptors. Because the ER stress pathway is a primary regulator of protein folding and synthesis in cells, understanding the impact of cannabinoids on the ER stress pathway is of vital importance for development of effective therapeutic agents.

## Conclusions

Cannabinoids have emerged as prominent modulators of diverse physiological and pathological processes. As such, efforts are underway to examine the efficacy of cannabinoid agonist and antagonists as therapeutic agents (McPartland et al., 2014; Russo, 2016; Toguri et al., 2016; Zhou et al., 2016). Cannabinoids primarily exert their effects through the cannabinoid receptors (CB1 and CB2); however, other receptors and molecular targets are now known to be important for their activity. The endocannabinoid system (ECS), which is composed of cannabinoids, cannabinoid receptors, and molecules involved in cannabinoid synthesis, uptake, and degradation, was initially described in the CNS. As a consequence, mechanisms of cannabinoid activity in the CNS are well-characterized compared to what is known about cannabinoid activity in cancer. This review sought to describe the cannabinoid receptor-independent actions of cannabinoids in systems where cannabinoid activity was both well- and poorly-defined. The studies examined herein indicate that, in the CNS, the activity of cannabinoids could be regulated by ion channels, enzymes, transporters, and receptors other than CB1 and CB2. In cancer cells, the CB receptor-independent activity of cannabinoids was found to be mediated by receptors, ion channels, lipid rafts, enzymes, and cellular stressors. Interestingly, in both the CNS and in cancer, several components of the ECS were important for CB1/CB2 receptor-independent activity including GRP55, TRP channels, FAAH, and MAGL. The dependence of cannabinoid activity on these common targets in dissimilar systems reinforces the importance of the ECS in cannabinoid signaling and activity.

Studies described in this review also reveal cannabinoid targets that are unique to the CNS and cancer. In the CNS, cannabinoids generated different biological responses through modulation of opioid, serotonin, adenosine, amino acid, and cholinergic receptors. Opioid, serotonin, NMDA, and GABA receptors have been implicated in cannabinoid-mediated modulation of cancer pain and chemotherapy-induced emesis (Maccarrone et al., 2009; Gu et al., 2011; Khasabova et al., 2011; Bolognini et al., 2013; Higgins et al., 2013; Ward et al., 2014). Future investigations should determine the role of these receptors in cannabinoid-induced tumor cell death—particularly in CNS malignancies. Research in cancer cells also demonstrated that the activity of cannabinoids could be mediated by lipid rafts, ER stress, oxidative stress, and enzymes. The involvement of lipid rafts, ROS and enzymes (COX-2 and phosphatases) in CNS cannabinoid signaling have been described in a few studies (Cannich et al., 2004; Maccarrone et al., 2009; Oddi et al., 2012; Penumarti and Abdel-Rahman, 2014b; Colín-González et al., 2015); however, the impact of the ER stress pathway on cannabinoid activity in the CNS remains an open question.

The diversity of cellular targets for cannabinoid ligands helps to explain the wide range of physiological responses to cannabinoid drugs. This range of efficacies also suggests that opportunities for employing cannabinoid-based therapies have only just begun to be explored. More needs to be learned about CB1/CB2-independent targets of cannabinoids to identify off-receptor effects, mechanisms of tolerance/ resistance and to explain unanticipated outcomes. In addition, by examining common and distinct cannabinoid targets in different biological systems, unique mechanisms of drug action can be uncovered. Among these biological systems are, notably, animals more primitive than chordates that lack the classical cannabinoid receptors (Elphick, 2012). Despite absence of these receptors, there is significant literature documenting behavioral effects in these organisms (reviewed by Soderstrom, 2009). To the extent that some signaling systems are conserved across vertebrate and invertebrate species (e.g., serotonergic signaling, Tierney, 2001) these behavioral effects may involve subsets of the array of non-CB1/CB2 targets reviewed here. Thus, invertebrate species, without interfering classical cannabinoid receptors (and therefore representing “clean” systems), hold promise for studying mechanisms of off-receptor cannabinoid effects.

## Author contributions

KS and ES contributed equally to this work. KS, ES, and RV contributed to the conception and design of the review, the acquisition of data, and the analysis and interpretation of the data. All authors participated in drafting and revising the manuscript. All authors approved of the final version of the submitted manuscript.

### Conflict of interest statement

The authors declare that the research was conducted in the absence of any commercial or financial relationships that could be construed as a potential conflict of interest.
